# Age Differences of the Hierarchical Cognitive Control and the Frontal Rostro–Caudal Functional Brain Activation

**DOI:** 10.1093/cercor/bhab382

**Published:** 2021-11-02

**Authors:** Zai-Fu Yao, Shulan Hsieh

**Affiliations:** Brain and Cognition, Psychology Research Institute, University of Amsterdam, 1001 NK Amsterdam, The Netherlands; Graduate Institute of Sports Training, College of Kinesiology, Tianmu Campus, University of Taipei, Taipei City 11153, Taiwan; Department of Psychology, College of Social Sciences, National Cheng Kung University, Tainan City 70101, Taiwan; Institute of Allied Health Sciences, College of Medicine, National Cheng Kung University, Tainan City 70101, Taiwan; Department of Public Health, College of Medicine, National Cheng Kung University, Tainan City 70101, Taiwan

**Keywords:** aging, cognitive control, fMRI, frontal lobe, working memory

## Abstract

Age-related differences in the functional hierarchical organization of the frontal lobe remain unclear. We adopted task-related functional magnetic resonance imaging (fMRI) to investigate age differences in the functional hierarchical organization of the frontal lobe. Behavioral results report both reaction time and efficiency declined as the levels of abstraction increased in the selection of a set of stimulus–response mappings in older adults compared with young adults. fMRI findings suggest trends of the hierarchical organization along the rostro–caudal axis in both groups, and brain–behavior correlation further suggests neural dedifferentiation in older adults when performing at the highest level of control demands experiment. Behavioral performances and age difference overactivations at the highest level of control demands were both associated with working memory capacity, suggesting the working memory capacity is important for processing the highest task demands. Region-of-interest analysis revealed age differences in brain overactivation and common activation across experiments in the primary motor cortex, parietal lobule, and the fusiform gyrus may serve as shared mechanisms underlying tasks that are required for the selection of stimulus–response mapping sets. Overall, older adults reflect maladaptive overactivation in task-irrelevant regions that are detrimental to performance with the highest control demands.

## Introduction

Frontal lobes are typically thought of as crucial brain regions for executive functions (Miyake et al. 2000; Alvarez and Emory 2006; Anderson et al. 2011; Badre and Nee 2018), especially for the cognitive control of goal-directed behavior (Badre 2008). The frontal lobe is the cerebral cortex that covers the anterior part of the frontal lobe; this brain region has been implicated in planning complex cognitive behavior, personality expression, decision making, and moderating social behavior (Bicks et al. 2015). Although the importance of frontal lobes is self-evident, its functional organization remains unclear. Badre and D’Esposito (2009) proposed the rule of abstraction models based on observations across a range of neuroimaging and neuropsychological studies of a functional gradient along the rostro–caudal axis of the frontal lobe, whereby progressively anterior subregions of the frontal lobe are associated with higher-order processing requirements of planning and selection of action (Badre 2008; Badre and D’Esposito 2009). To support their notion, Badre and D'Esposito (2007) conducted series of experiments to examine whether the frontal lobe is relatively homogeneous in function or whether specialization and organization along the rostro–caudal axis in both healthy individuals (Badre and D’Esposito 2007) and patients with damage to the frontal lobe occur (Badre et al. 2009). According to their hypothesis, the most caudal region is associated with processing information about the concrete stimulus, whereas more anterior frontal lobe activity is seen when behavior is instructed by the abstract stimulus. Their results showed that the frontal lobes are organized along their rostro–caudal axis to support hierarchical cognitive control (Badre and D’Esposito 2007; Badre et al. 2009). Despite fruitful evidence suggesting the frontal lobes are necessary for cognitive control at all levels of abstraction, whether aged frontal lobes are still activated hierarchically with different levels of abstract rules remains unclear. Although Badre’s fMRI competition experiments provide the first evidence that functional organization processes hierarchically along the rostro–caudal axis of the frontal lobe in both healthy individuals (Badre and D’Esposito 2007) and patients with damage to the human frontal lobe (Badre et al. 2009), whether these observations can be seen in an aging population remains to be investigated.

As our brains age, we tend to experience cognitive decline, which may cause the lack of capability to recruit specific areas or an attempt to compensate for the aging process (Deary et al. 2009; Cabeza et al. 2018; Cole et al. 2019). The attempt to compensate for the mismatch between cognitive processing and task demands by increasing neural activity or connectivity may lead to enhanced cognitive processing (successful compensation) or it may lead to no change in performance or even worse performance (unsuccessful compensation). For instance, age-related increases in activity in the frontal lobe and connectivity have been attributed to compensatory brain activity (Stuss and Knight 2009; Nyberg et al. 2010). Nonetheless, these findings question whether efficiency for processing competition among the abstractness of the representation progresses with age, specifically, whether hierarchical cognitive control and the rostro–caudal functional gradient organization of the frontal lobes process are similar or different between different age groups. Moreover, it is well-documented that older adults recruit other brain regions while performing a wide variety of cognitive tasks compared with those recruited by younger adults (Gutchess et al. 2005; Davis et al. 2008; Cabeza and Dennis 2012; Morcom and Henson 2018). However, it is unclear how such age-related overactivation involved at different levels of control demand abstract representations and whether this overactivation is compensatory.

Previous studies have shown that overactivation in frontal brain regions for older adults while performing tasks (Davis et al. 2008; Reuter-Lorenz and Cappell 2008; Park and Reuter-Lorenz 2009; Reuter-Lorenz and Park 2014; Nashiro et al. 2018; Qin and Basak 2020) might reflect compensatory brain activity to delay cognitive decline in older participants (Cabeza et al. 2018). In regards to age-related increases in PFC activity, two well-known theories of the pre-frontal cortex (PFC) compensation are posterior–anterior shift with aging (PASA) as described by Davis et al. (2008) and hemispheric asymmetry reduction in older adults (HAROLD) as described by Cabeza (2002) and Koen and Rugg (2019). For PASA, there is evidence of an age-related reduction in functional connectivity involving posterior brain regions coupled with an age-related increase in functional connectivity with PFC regions (Grady et al. 2003; Daselaar et al. 2006; St. Jacques et al. 2009). As for HAROLD, evidence that aging is not only associated with more bilateral activation patterns but also with an increase in functional connectivity to homologous regions in the two hemispheres can be found. An alternative explanation for the increase in brain activity in the frontal lobe is more related to reduced functional efficiency or specificity in the aged brain (such as the dedifferentiation hypothesis; Koen and Rugg 2019) than to compensation. This dedifferentiation hypothesis put forth that task-evoked neural activity in the brain regions becomes less selective with increasing age. This interpretation of neural dedifferentiation is a consequence of the age-related decline in functional specialization of brain regions in older rather than in younger adults such that brain regions specialized for a single cognitive function in younger adults are adopting multiple cognitive functions for completing the task (Dennis and Cabeza 2011; Reuter-Lorenz and Park 2014; Koen and Rugg 2019). These types of overactivation have been argued to be either compensatory to older adults’ performance, indicated by positive brain–behavior correlations (Cabeza 2002; Davis et al. 2008; Cabeza and Dennis 2012; Basak et al. 2018), or are maladaptive to performance as indicated by a negative brain–behavior correlation (Steffener et al. 2014; Nashiro et al. 2018). Specifically, the observation of overactivations is interpreted as a compensatory mechanism if a significant, positive, brain–behavior relationship with task performance is reported (Cabeza et al. 2018). Our goal was to test whether increased frontal lobe activity in older adults reflects compensation or nonselective recruitment (e.g., neural dedifferentiation).

Furthermore, if maladaptive types of overactivation are observed in older adults, activation of such task-irrelevant regions may add detrimental noise to the memory system and contribute to worsened updating (Qin and Basak 2020). Previous studies have shown that older adults exhibit deficient working memory performance that is related to a selective deficit in inhibiting sensory processing related to irrelevant stimuli (Gazzaley et al. 2005; Chadick et al. 2014). Importantly, such a decline in suppressing irrelevant stimulus processing is associated with a decrease in activity in the frontal lobe in addition to a decrease in functional connectivity between the sensory cortex and the frontal lobe (Chadick et al. 2014). Recent studies on age-related overrecruitment in the frontal lobe depending on memory load may be attributed to limited-resource functional compensatory mechanisms (Cappell et al. 2010; Qin and Basak 2020). Accordingly, older adults may exhibit deficient working memory performance due to overloading limited memory stores with irrelevant information. Therefore, it is worth testing the brain–behavior relationship between age differences in brain activity and behavioral performance changes by exploring the aging effects on the processing of different levels of behavioral abstraction and its relationship to working memory.

## Objectives and Hypotheses of the Present Study

This study aims to examine whether functional organization process hierarchically along the rostro–caudal axis of the frontal lobe can still be seen in older adults as observed in younger adults reported by prior research (e.g., Badre and D’Esposito 2007). We adopted the identical protocol described by Badre and D'Esposito (2007) to investigate the functional gradient of the frontal lobe in four experiments. Behaviorally, we hypothesized that older participants have worse performance across all conditions as reaction time (RT) slowing due to aging is commonly observed. RT slowing first occurs in sensorimotor regions that are typically involved in how the individual forms a response. However, as difficulty levels increase, the ceiling effects make it harder to distinguish between groups since young adults also process longer RTs at the most difficult levels. Therefore, we hypothesized that a lower level of difficulty manipulation associated with the sensory process may be affected by the age effect.

At the neural activity level, we first aimed to examine the patterns of fMRI activation in young adults to determine whether a functional gradient along the rostro-caudal axis of the frontal lobe could be replicated, whereby progressively anterior subregions of the frontal lobe are associated with higher-order processing requirements of more abstract action planning. The second goal was testing whether efficiency at processing competition among the abstractness of the representation declines with increasing age. Specifically, we aimed to test whether the rostral-to-caudal functional gradient of the frontal lobe is disrupted by the aging processing. Our goal was to demonstrate whether functional specialization and organization in the frontal lobe would have different neural processing strategies for the older group. Therefore, it was determined which cortical surface vertices were related to the regions that were most parametrically correlated so that competition from the lowest to the highest levels of difficulties in task performance of older and young groups could be determined. Moreover, whether overrecruitment of frontal brain activity evoked by a cognitive control task reflects compensatory responses (e.g., compensatory theory) or a deterioration process due to age was tested. The HAROLD model (Cabeza 2002; Koen and Rugg 2019) described that overrecruitment of the bilateral PFC in older adults in comparison to young adults whose PFC is activated in the lateralized brain reflects a compensatory mechanism in order to keep up with the equivalent task performance. Whether this observation can be explained by neural dedifferentiation, especially with task performance at multiple levels of abstract representations (Koen and Rugg 2019), was examined. These hypotheses were tested by observing whether age-related overactivation of the fronto–parietal regions interacts with different degrees of cognitive control demands involved in a task. If an interaction had occurred, the compensatory hypothesis would be a more reasonable choice. According to a recent study (Qin and Basak 2020), the relationship between age differences in overactivation and task performance suggests different possible explanations. Specifically, a positive correlation between activation based on age difference in activation of ROIs and task performance would indicate that such activations are compensatory with respect to task performance. A negative correlation between activation and task performance would suggest that such activation is detrimental to task performance (Qin and Basak 2020). Finally, whether behavioral performances and age differences of brain activity processing at levels of cognitive controls are associated with working memory capacity was also tested.

## Materials and Methods

### Participants

A total of forty-five participants were enrolled in this study. These participants were grouped into older and younger groups based on their chronological age of demographic information. All participants with normal or corrected-to-normal vision were recruited for participation in four mini experiments in an fMRI scanning session. Three older participants failed to complete the fMRI scanning sessions due to exhaustion. Two of the older participants were excluded from subsequent fMRI analysis due to technical difficulties (i.e., preprocessing quality failed to meet the criteria and data export issues) with their scans (e.g., temporal signal loss). All MRI images were inspected visually. Specifically, MRI data with obvious motion artifacts are excluded from the analyses. Two young adults were excluded from subsequent analysis because of motion artifacts (FD = 0.25 mm) (Power et al. 2012). We finally included 38 right-handed healthy adult participants for the following analysis. Informed consent was obtained from participants following procedures approved by the Committee for Governance Framework for Human Research Ethics at the National Cheng Kung University. Participants were rewarded of New Taiwan dollar (NTD) 1000 for their participation in MRI sessions. All participants reported no history of brain injury, neurological disease, mental disorder, and clinically diagnosed hypertension. The demographic information of participants was reported in Table 1 and Supplementary Table 1.

**Table 1:** Demographic information for participants included in this study

	Old	Young	*t*	df	*P*-value
N	19 (f = 7; m = 12)	19 (f = 8; m = 11)	N/A	N/A	N/A
Age	67.42 (±5.98)	24.31 (±5.23)	23.623	36	1.645e−23^*^
MoCA	28.7 (±1.27)	29.4 (±0.83)	−1.807	36	0.079
BDI-II	4.73 (±5.17)	8.26 (±7.89)	−1.629	36	0.112
Education (yr)	14.632 (±2.49)	16.789 (±1.78)	−3.064	36	0.004^*^
WM span	22 (±3.48)	27 (±1.44)	−6.377	36	2.185e−7^*^

^*^N = number of participants; f = female; m = male; MOCA = The Montreal Cognitive Assessment; WM = working memory; BDI-II = Beck Depression Inventory-II.

### The Montreal Cognitive Assessment (MoCA)

The MoCA (Nasreddine et al. 2005) is a cognitive screening test designed to assist clinical professionals to identify mild cognitive impairment and early signs of Alzheimer’s disease. MoCA scores range between 0 and 30. A score of 26 or over is commonly assumed to be normal. In this study, a validated Taiwanese version of MoCA (Tsai et al. 2012) was used to screen the participants.

### Auditory–Verbal Digit Span: Working Memory

Digit span (Szczepkowski and Demakis 2017) is the most widely used measure of verbal working memory capacity. In this study, two different types of recall methods were administered: “forward” digit span, needing simple recall of a serial of digits (word for word) to be remembered, and “backward” digit span, needing to verbally repeat a given serial of digits (word for word) in reverse order. The behavioral performance was calculated by summing the total correctly recalled trials. The higher the scores, the better the working memory capacity (Hilbert et al. 2015).

### Beck Depression Inventory-II (BDI-II)

BDI-II is a short, criteria-referenced assessment for measuring depression severity (Li et al. 1975). Higher total scores indicate more severe depressive symptoms (Steer et al. 1999). The total score of 0–13 is considered a minimal range, 14–19 is mild, 20–28 is moderate, and 29–63 is severe.

### Behavioral Tasks and Experimental Procedures

In this experimental design, we aim to test whether the rostral-to-caudal functional gradient of the frontal lobe is disrupted by aging processing. We adopted the identical protocol as Badre and D'Esposito (2007) to investigate the functional gradient of the frontal lobe in four experiments. Participants were tested on four mini experiments that were designed to test progressively higher degrees of difficulties with the varied competition. These experiments were adapted from a previous study (Badre and D’Esposito 2007). Specifically, from concrete to abstract, four fMRI experiments manipulated the abstractness of stimulus–response rules over four levels of competition (Supplementary Figs 1–4). Each subsequent level increased the contextual contingencies to be traversed to select a response (color–feature–finger, color–dimension–feature–finger, episode–color–dimension–feature–finger). Experiments had one, two, or four hierarchical levels of rule complexity, which served as a parametric variable approximating cognitive demand in the data analysis to identify regions specifically involved in cognitive loading at each rule order. The procedure of each experiment consisted of blocks with manipulation of the abstractness of stimulus–response rules over three hierarchical levels of rule complexity.

Before performing each block, participants were shown all the color mappings that they would encounter for that task, one block at a time. The mappings were covered, and the participants have performed one practice block with the mapping set that they had just memorized. The practice block was identical to the experimental setting with eight trials. During initial training outside the scanner, participants were taught the mappings they would encounter in the fMRI scanning session and then practiced both outside and inside the scanner.

In each trial of blocks, an instruction screen cued the participant which stimulus–response rule mappings were relevant (until response) before the outset of each block. Specifically, at the beginning of each cueing task, participants were shown the size, orientation, as well as texture of each target object as an example for reference. Individual trials were separated by a jittered null fixation interval (0–4 s). Instruction screen of color cue for each block (10 s per block). Participants were then shown a colored square as a visual contextual cue with a target object on the screen for 3.9 s followed by a 100-ms noise mask presented one at a time. Participants could make a response after the target is present on the screen. The time window of response allowed for a maximum of 2 s to respond. The reasons for this arrangement were to make sure the older adults have enough time to respond. Trials within a block were separated by jittered fixation-null events (0–4 s).

In the response experiment (Supplementary Fig. 1), participants responded based on stimulus–response associations (color–finger). Based on a learned color-to-response mapping from instruction, subjects made a button response on an fMRI manual response pad under their right hand depending on the color of the presented square. In each subsequent block, only four color-to-response mappings were relevant for each block. During 1-Response blocks (R1), all four colors mapped to a single response. During 2-Response blocks (R2), there were two of the four colors mapped to each of two responses, producing response competition. During 4-Response blocks (R4), each of the four colors uniquely mapped to one of the four responses, producing the greatest response competition. As these responses were mapped to colored squares only [same dimension (e.g., color) and feature (e.g., colors)], the competition was minimal.

In the feature experiment (Supplementary Fig. 2), participants responded based on stimulus–response associations (color–feature–finger). The feature experiment manipulated feature competition by varying the number of specific textures of an object that could map to a given response. Participants were presented with a series of colored squares one at a time (3900 ms, followed immediately by a noise mask of 100 ms), and each square contained a single object. Trials were separated by a jittered null fixation interval (0–4 s).

In the dimension experiment (Supplementary Fig. 3), the series of colored boxes each contained two objects that each varied along four dimensions (texture, shape, size, and orientation) from trial to trial. The participants were required to decide whether the objects matched along only one of those dimensions on each trial. The relevant dimension was cued by the colored box. Competition increased with the number of alternative dimensions for a given block increasing from one (low) to two (mid) to four (high).

The context experiment (Supplementary Fig. 4) was identical in terms of the task instructions to the dimension experiment, except that two dimensions were always relevant across all blocked conditions. Moreover, in the context task, a given color cue could map to different dimensions on different blocks (in the dimensioning task, a given color is always mapped to one dimension). Thus, in the context task, it is necessary to use information about the current temporal frame (the current block or the most recent instructions) to select the appropriate mapping for a given color cue. Thus, the competition was manipulated by varying the frequency across blocks that a given color cue (the context) mapped to a specific dimension. Certain color-to-dimension mappings were relevant for 100% of the blocks in which that cue was encountered, other color-to-dimension mappings were relevant for 50% of blocks in which that color was encountered, and other color-to-dimension mappings were relevant on only 25% of blocks in which that color is encountered. In the latter two cases, determining which color-to-dimension mapping is currently relevant requires the selection of a particular color-to-dimension mapping based on the instructions of the current block. In this way, as the frequency of a given color-to-dimension mapping decreases, uncertainty or competition with other mappings increases and so selection of the currently relevant mapping requires more control.

### Experimental Apparatus

All experiments were coded, presented, and collected in Presentation (Version 18.0, Neurobehavioral Systems, Inc., Berkeley, CA, www.neurobs.com), which is frequently used as task creation and presentation software application in fMRI studies. Across all experiments, visual cues consisted of centrally presented colored squares (subtending 68° of visual angle). The fixation target was designed as a previous study (Thaler et al. 2013) suggested for combined low dispersion and microsaccade rate to improve optimal stability of ocular fixation across experiments. For a given experiment, one of two distinct color sets of eight colors was used twice across the four experiments, and the order was counterbalanced across subjects. Object stimuli used in the feature, dimension, and context experiments consisted of grayscale, 3-D shapes. Objects were images labeled with permission for reuse with modification from objects—Textures.com (https://www.textures.com/) designed to be unfamiliar, difficult to name, and without real-world counterparts. Piloting determined that these feature variations in these objects were easily distinguishable. The four mini experiments (response, feature, dimension, and context) were tested in an fMRI scanning session. Experiments were counterbalanced for order across subjects, while the order of experiments was performed as follows (based on Badre and D’Esposito 2007): The context experiment was always performed first, the dimension experiment was always performed in the session after the context experiment, the response experiment was performed at third after the dimension, and the feature experiment was performed at last. The rest of each experiment was determined by the participant.

In each block, thirty-two trials consisted of twenty-four trials for the target event, and eight trials for the fixation-null event (25%) were included in each run, respectively. The trials have scheduled the order and timing of events for event-related fMRI experiments using NeuroDesign (http://neuropowertools.org/design/start/) across participants. To minimize discomfort due to a long period of scanning in older participants, we shortened the number of trials across experiments. For the context experiment, participants performed 192 trials consisting of six blocks of mappings. The order of blocks, cycled twice, was a block of low-competition condition, followed by a block of high-competition condition, and finally by a block of midcompetition condition. The response, feature, and dimension experiments consisted of three blocks, and each competition condition (from low, mid, to high) was randomly presented for order across participants. Each block was self-paced after participants understood the instruction and were fully counterbalanced for an order for the response, feature, and dimension experiments. All participants were encouraged to respond as quickly and as accurately as possible on every trial. The specific color mappings, responses, and objects used in the tasks were counterbalanced across subjects and two-color sets were used to minimize confusion between tasks. Where applicable in each experiment, color cue, response, feature, and dimension switches were controlled for frequency across blocks of each condition. All combinations of colors and features in the feature experiment and colors and shapes in the dimension and context experiments were controlled across the competition and switching conditions.

### fMRI Acquisition

The imaging data for all four experiments were collected on a General Electric (GE) Discovery MR750 3 Tesla scanner (General Electric Medical Systems, Milwaukee, USA) using a 32-channel receive-only phased-array head coil in the Mind Research Imaging center at the National Cheng Kung University. High-resolution structural images were acquired with fast-SPGR consisting of 166 axial slices (TR/TE/flip angle 7.6 ms/3.3 ms/12°; a field of view (FOV) 22.4 × 22.4 cm^2^; matrices 224 × 224; slice thickness 1 mm). The functional EPI images were collected using an interleaved T2^*^ weighted gradient-echo planar imaging (EPI) pulse sequence (TR/TE/flip angle, 2000 ms/30 ms/77°; matrices, 64 × 64; FOV, 22 × 22 cm^2^; slice thickness, 4 mm; voxel size, 3.4375 × 3.4375 × 4 mm). A total of 75 volumes were acquired for each block; the first five were dummy scans and were discarded to avoid T1 equilibrium effects. Moreover, localizer scans (5 min) and distortion correction scans (B0 field map and a pair of spin-echo EPI scans with opposite phase-encoded directions) are also acquired. The visual stimulus was displayed using Presentation (Version 18.0, Neurobehavioral Systems, Inc., Berkeley, CA, www.neurobs.com) on an MSI GS65 Stealth laptop and was projected onto a screen that was viewed through a mirror attached to the head coil.

### Statistical Analysis—Behavioral

Average median reaction time (mRT) across all experiments and mappings were obtained for each participant. Delta reaction time (dRT) was the calculated difference between mRT and overall averaged reaction time in each mapping across all participants for taking reaction time distributions and individual differences into account (Wagenmakers and Brown 2007; Pratte et al. 2010). Inverse efficiency score (IES) (Townsend and Ashby 1978) was also calculated to deal with speed–accuracy tradeoffs in RT experiments. The higher the IES, the lower the performance accuracy. Group comparison between young and older adults was also conducted using the independent sample *t*-test (see Supplementary Tables 2–4). Pearson’s correlation analysis was also conducted to measure the statistical association between the behavioral performance of fMRI tasks and demographic measurements (see Supplementary Table 5). Additionally, we performed the Bayesian version of statistical tests to examine the strength of evidence in favor of our hypothesis by JASP (Version 0.14.1, https://jasp-stats.org/). Specifically, the Bayes factor (BF) provides an easily interpretable index of preference for one hypothesis over another that has advantages over traditional null hypothesis testing techniques (Keysers et al. 2020). It can be interpreted as a measure of the strength of evidence in favor of one theory among two competing theories. BFs are indices of relative evidence of one “model” over another. BF was computed as a Savage-Dickey density ratio (Makowski et al. 2019), which is also an approximation of a BF comparing the marginal likelihoods of the model against a model in which the tested parameter has been restricted to the point-null (Wagenmakers et al. 2010, 2018). For both the behavioral and following fMRI results, we report classical frequentist *P*-values and BFs, which provide a more conservative evaluation of the correlations. We also provide using the Bayesian independent *t*-tests with the BF for group comparison. We provide BF_10_ that is giving the evidence for alternative hypotheses over null hypotheses. BFs may be interpreted as proportional evidence for the presence or absence of an effect. For instance, a BF_10_ of five indicates that the data are five times more likely to occur under the alternative hypothesis than under the null hypothesis (van Doorn et al. 2021). In addition, we can interpret the BF categorically based on a grouping of evidence accumulated. For a detailed explanation of the Bayesian statistics and the BF, see van Doorn et al. (2021).

### Statistical Analysis—Structural Volumes

Structural images were analyzed with FSL-VBM (http://fsl.fmrib.ox.ac.uk/fsl/fslwiki/FSLVBM) (Ashburner and Friston 2000, 2001; Bookstein 2001) for optimizing voxel-based morphometry analysis. First, structural images were extracted from nonbrain tissues using BET and gray matter segmented before being registered to the MNI 152 standard space with nonlinear registration (Andersson et al. 2007). The thresholded images were extracted and averaged to generate a GM template for this study. After creating the GM template, GM images were nonlinearly reregistered to the template and adjusted for local contraction because of the nonlinear spatial transformation. The registered partial volume images were then adjusted for local contraction by dividing the Jacobian’s warp field. The adjusted segmented images were smoothed to sigma of 3 mm with an isotropic Gaussian kernel. Group comparison of gray matter differences between young and older adults was measured using permutation-based nonparametric testing (Winkler et al. 2014) incorporating threshold-free cluster enhancement (TFCE) with the significance of *P* value at <0.05 (Smith and Nichols 2009; Salimi-Khorshidi et al. 2011), family-wise error (FWE) was used for correcting multiple comparisons. To process structural images, we also derived voxel-wise gray matter nuisance regressors for each participant, which were used in the analysis of functional images to control for structural differences on the individual’s voxel level, following the method used by Oakes et al. (2007). After tissue-type segmentation using FSL’s automated segmentation tool (FAST) (Zhang et al. 2001), gray matter images were normalized into MNI 152 standard space and then demeaned across the participants. Further mediation analysis is conducted to verify whether the functional changes are the reflection of atrophy. For the mediation analysis, we used Mplus (Mangold 2017) emulation on JASP statistical software (https://jasp-stats.org/) (JASP Team 2020) to build a mediation path model without any latent variables. This estimated both the direct and indirect effects on age differences in functional changes across experiments. The model was estimated using maximum likelihood estimation and bootstrapping methods (Vinet and Zhedanov 2011). The significance of indirect effects was assessed with a 95% confidence interval. To estimate confidence intervals, we used a bias-corrected method with the percentile bootstrap estimation approach, which ran 1000 bootstrap iterations that were implemented (Hayes 2009). Adopting a two-tailed *P* < 0.05, we rejected the null hypothesis if the interval did not include zero. In particular, the rationale of the bootstrapping approach over other traditional approaches of mediation analysis is its improved sensitivity for estimating indirect effects (Demming et al. 2017). To interpret the results, if the CI included zero, we concluded that the indirect effect was not significant because zero suggests no relationship between the mediator and dependent variable. Conversely, the CIs that did not include zero suggested that there was a significant relationship (Kane and Ashbaugh 2017). Standardized coefficients are reported after the data were transformed to *z*-scores and before entry into the model (see Supplementary Table 6).

### Statistical Analysis—fMRI

All MR image data analyses were conducted using FSL (version 5.0.10) (Jenkinson et al. 2012). Computation was performed on a Dell PowerEdge server with a Linux system (Debian/Ubuntu) (32 Cores/64 Threads and 32 GB Memory). All raw images output from the scanner were converted by conversion tool “dcm2niix” to NIfTI format (https://www.nitrc.org/plugins/mwiki/index.php/dcm2nii:MainPage). In FSL, analyses were carried out using the FMRI Expert Analysis Tool (FEAT, v6.00). Brain extraction was performed on both the functional and structural data. The Brain Extraction Tool (BET) (Smith 2002) was applied to each structural image from the command line before preprocessing, and for functional data with the BET option within the Prestats module of FEAT, time-series statistical analysis was carried out using FILM with local autocorrelation correction (Woolrich et al. 2001, 2004). Specifically, preprocessing steps included MCFLIRT for motion correction (Jenkinson et al. 2002), nonbrain removal, spatial smoothing with a Gaussian kernel (5 mm), and high-pass temporal filtering with Gaussian-weighted least-squares straight-line fitting (sigma = 50s) (Smith et al. 2004). Field maps were generated (Duong et al. 2020) for distortion correction. Specifically, we calculate the difference in phase between the two images is proportional to the difference in echo times and the B0 inhomogeneity. The field map is calculated by taking the difference between the two-phase images and dividing that by the echo time difference using TOPUP (https://fsl.fmrib.ox.ac.uk/fsl/fslwiki/topup). Boundary-based registration (Greve and Fischl 2009) was performed in a two-step procedure using FLIRT (FMRIB’s Linear Registration Tool) (Jenkinson and Smith 2001): First, field map–corrected EPI images were registered to the high-resolution brain T1-weighted structural image (6-parameter affine transformation). Second, the transformation matrix (12-parameter affine transformation) from the T1-weighted image to the Montreal Neurological Institute (MNI) template brain was estimated. This allowed for transforming the EPI images to the standard MNI template brain.

As each mini experiment constituted an independent dataset, separate statistical models were constructed for each of the four experiments (i.e., response, feature, dimension, and context) under the assumptions of the general linear model. Epochs corresponding to each block of trials within a session were included in the statistical model along with regressors for the instruction periods at the beginning of each block. For each analysis, at the first level, a separate .fsf file was created for each scanning session. Runs were then combined as part of a second-level fixed-effects model, yielding results that were subsequently inputted into a group analysis. Furthermore, blocks of each mapping condition were weighted as a parametric regressor in the model. Statistical effects were estimated using a subject-specific fixed-effects model, with session-specific effects and low-frequency signal components (<0.01 Hz) treated as confounds. Linear contrasts between target trials and null trials were used to obtain subject-specific estimates for each effect. These estimates were entered into a third-level group comparison analysis treating subjects as a random effect, using GLM model for two-sample unpaired *t*-test against a contrast value of zero at each voxel. These data were then cluster corrected so that only clusters with a *Z*-score of at least 3.1 and a *P* value lower than 0.001 survived.

All GLM analyses were carried out in the following steps (Beckmann et al. 2003). First, blood oxygenation level-dependent (BOLD) time series were prewhitened with local autocorrelation correction. A first-level FEAT analysis was carried out for each run of each subject. Second, a second-level (subject-level) fixed-effect (FE) analysis was carried out for each subject that combined the first-level FEAT results from different runs using the summary statistics approach. Finally, a third-level (group-level) mixed-effect (ME) analysis using FSL’s FLAME module (FMRIB’s Local Analysis of Mixed Effects) was carried out across subjects by taking the FE results from the previous level and treating subjects as a random effect (Woolrich et al. 2004). Specifically, analyses in the third level, the main effect of each experiment, were contrasting to obtain the group BOLD response for each experiment. The significance threshold of group-level results was set to *P* < 0.05 with family-wise error rate (FWE) corrected and cluster-wise inference with a *P* ≤ 0.001 uncorrected cluster forming threshold. To understand the main and interaction effects of the fMRI analyses better, we extracted the parameter estimates of the GLM for each participant. For each cluster of the main and interaction effects, a mask was created. For each mask, mean parameter estimates were extracted for each condition in each group of participants. Task-related effects will be estimated according to the general linear model at each voxel. Group effects will be estimated using a random-effects analysis to highlight brain regions activated in common in all subjects along with group-related differences, and the statistical maps will be corrected for false discovery rate (FDR). All reported whole-brain results were corrected for multiple comparisons. We first identified clusters of activation by defining a cluster-forming threshold of the *z* statistic (*z* > 3.1, *P* < 0.001) (Worsley 2012; Woo et al. 2014; Eklund et al. 2016). Then, an FWE corrected *P* value (*P* < 0.05) of each cluster based on its size was estimated using Gaussian random field theory (Worsley et al. 1992). In addition, we performed a nonparametric permutation test using the randomize function in FSL (threshold-free cluster enhancement or TFCE option (Smith and Nichols 2009) to identify significant clusters of activations on the contrasts reported in the study. All analyses conducted were adjusted for educational experiences. Based on results from the whole-brain analysis, we then use an independent and unbiased regions-of-interest (ROI) approach for confirming parametric effects on each level of mapping across independent experiments. Blocked parametric effects obtained in the group-level voxel-based contrasts between groups were subsequent with ROI analyses. Group functional ROI analysis was used to verify the characteristic event-related signal change in voxels identified by a prior block-level contrast. Moreover, ROI analysis based on prior findings with identical tasks (Badre and D’Esposito 2007) was also analyzed. ROI has then created a sphere mask based on significant neighboring voxels within a 5-mm radius around a chosen maximum *z*-statistic from the blocked parametric contrast at each level of mapping of experiments. We applied the Harvard–Oxford subcortical probabilistic atlas thresholded at 25% probability when performing the ROI analysis for reducing the effects of outliers. We then extracted the mean contrast of parameter estimates within the mask from each participant and used it for further statistical analysis. Moreover, seed-based correlation analysis between age difference in activation of ROIs and behavioral performance (i.e., working memory capacity and mRT difference scores (dRT)) for each experiment across all participants was also conducted (Supplementary Table 7–9). According to a recent study (Qin and Basak 2020), the relationship between age differences in overactivations and task performance indicates different possible explanations (Koen and Rugg 2019). Specifically, a positive correlation between activations from age difference in activation of ROIs and task performance would indicate that such activations are compensatory to task performance. A negative correlation between activations and task performance would suggest that such activations are detrimental to task performance.

## Results

### Behavioral Performances

**Figure 1:**
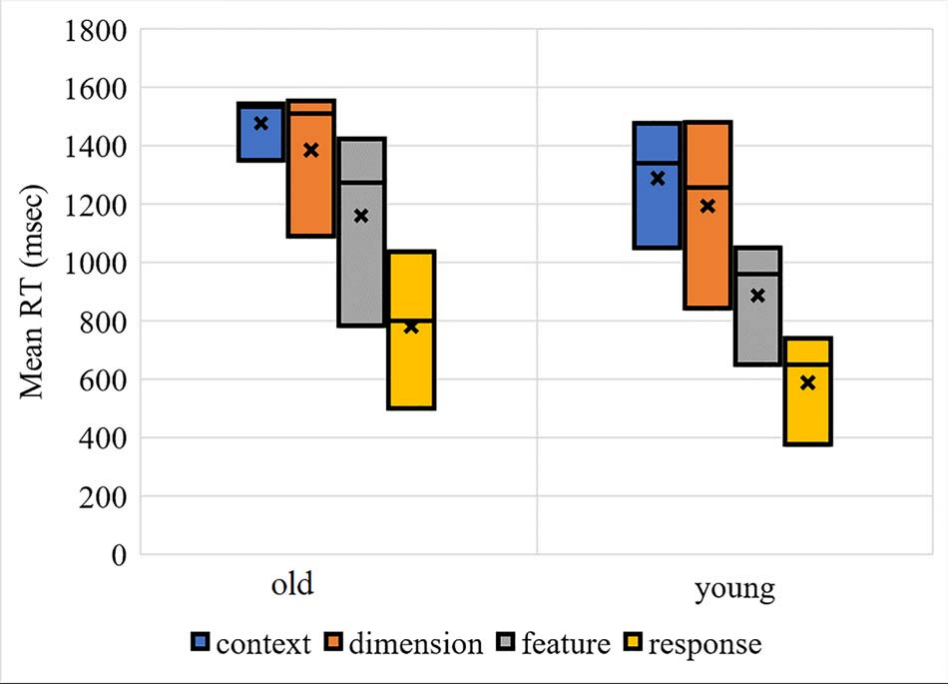
A box and whisker plot for average RT performance across four fMRI experiments. Generally, increases at higher mapping conditions are evident across all correct trials except mapping at midlevel of context experiment; RT = average median reaction time; msec = millisecond.

Average mRT and IES on mappings between young and older adults were reported as follows. In RT measures, significant age differences were observed on all mappings except for the most demanding mappings on context (*t*(36) = 0.712, *P* = 0.481) and dimension (*t*(36) = 0.28, *P* = 0.781) experiments. Average RT from the four fMRI experiments was reported in Figure 1. Moreover, in the IES measures, significant age differences similar to behavioral RT performances were observed across all competition except for the most demanding mappings on dimension (*t*(36) = −0.626, *P* = 0.535) (for detail, see Supplementary Tables 2–4). Correlation coefficient analysis shows significant linear relationship between IES scores across experiments and working memory span; specifically, working memory span is negatively correlated with IES scores (Supplementary Table 5) of response experiments (R1 (*P* = 0.03, *r* = −0.352), R2 (*P* = 0.029, *r* = −0.355), R4 (*P* = 0.01, *r* = −0.414)), feature experiments (F4 (*P* = 4.611e−5, *r* = −0.611)), dimension experiments (D1 (*P* = 0.004, *r* = −0.46), D2 (*P* = 0.001, *r* = −0.502)), and context experiments (C4 (*P* = 5.945e−5, *r* = −0.604)) (Supplementary Table 5). Moreover, correlation coefficient analysis shows significant linear relationship between RT performance of the task across experiments and working memory span; specifically, working memory span is negatively correlated with RT performance of response experiments (R1 (*P* = 0.038, *r* = −0.338), R2 (*P* = 0.01, *r* = −0.415), R4 (*P* = 7.747e−4, *r* = −0.522)), feature experiments (F2 (*P* = 0.047, *r* = −0.325), F4 (*P* = 0.001, *r* = −0.512)), dimension experiments (D1 (*P* = 0.009, *r* = −0.419), D2 (*P* = 0.043, *r* = −0.331)), and context experiments (C2 (*P* = 0.047, *r* = −0.324)) (Supplementary Table 10).

### Structural Differences between Older and Young Adults

We also investigated whole-brain voxel-wise differences in gray matter (GM) volume in both young and older adults, to determine whether the age-related structural GM changes at the individual level modulate the task-evoked functional brain activations. Voxel-based morphometry (VBM) (Ashburner and Friston 2000, 2001; Bookstein 2001) analyses revealed that young adults had a greater overall GM volume (754.04 ± 34.23 mm^3^) than older adults (690.1 ± 41.64 mm^3^) (*t*(36) = 2.339, FWE corrected *P* = 0.009, Cohen’s *d* = 1.463). Specifically, the most prominent age differences of GM volume differences were in the superior parietal lobule, superior frontal gyrus, middle temporal gyrus, superior temporal gyrus, and frontal pole after adjusting for education levels (see Supplementary Table 11).

### fMRI Results

**Figure 2:**
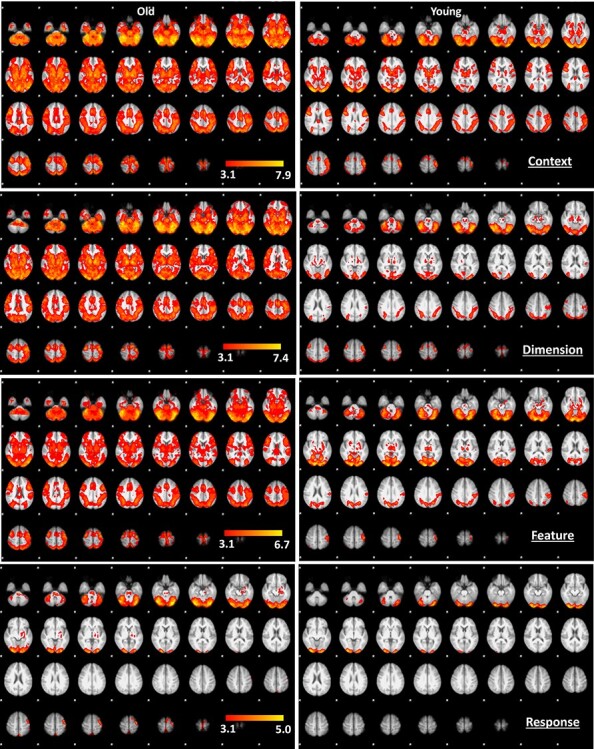
Task activation for parametric effects across four experiments in young and older adults. *Z* (Gaussianized *T*/*F*) statistic images were thresholded using clusters determined by *Z* > 3.1 and a (corrected) cluster significance threshold of *P* = 0.001.

**Figure 3:**
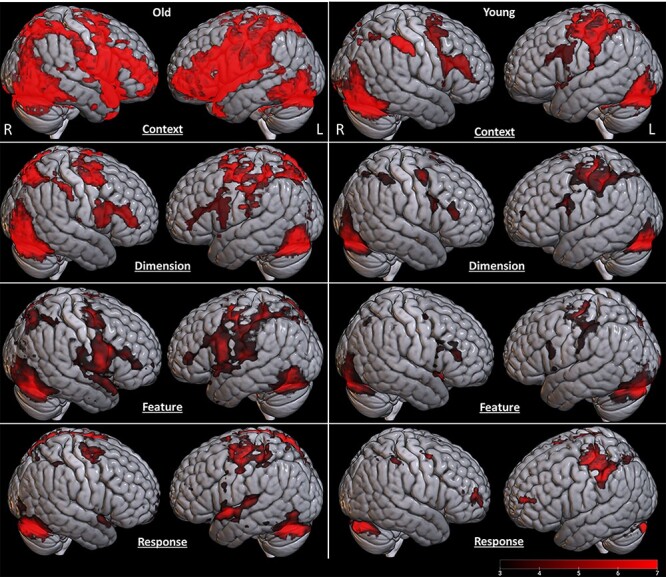
Task activation for parametric effects across four experiments in young and older adults. *Z* (Gaussianized *T*/*F*) statistic images were thresholded using clusters determined by *Z* > 3.1 and a (corrected) cluster significance threshold of *P* = 0.001.

**Figure 4:**
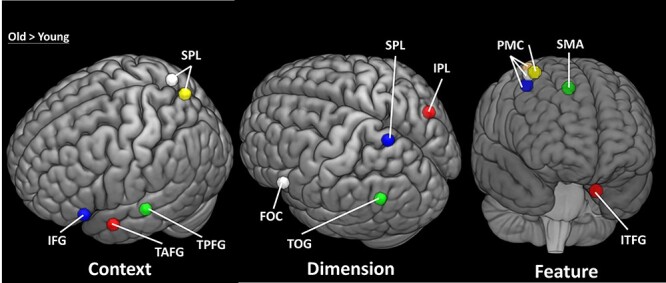
Age differences of BOLD response in contrast with older and young adults across experiments (target > null trial). IFG = inferior frontal gyrus; TAFG = temporal anterior fusiform gyrus; TPFG = temporal posterior fusiform gyrus; SPL = superior parietal lobule; FOC = frontal orbital cortex; TOG = temporal occipital gyrus; IPL = inferior parietal lobule; PMC = premotor cortex; SMA = supplementary motor cortex; ITPFG = inferior temporal fusiform gyrus.

Group BOLD response changes to mapping blocks for each experiment were reported in Figures 2 and 3. All whole-brain fMRI results were based on cluster-level inference and FWE corrected for multiple testing at *P* < 0.05. Two procedures were implemented. First, for cluster-level inference using Gaussian random field theory, we used *z* > 3.1 (*P* = 0.001) as the cluster-forming threshold (Eklund et al. 2016). Second, for cluster-level inference based on the nonparametric permutation test, we used the threshold-free cluster-enhancement procedure (Smith and Nichols 2009). Group comparison results showing significant clusters of parametric activation in each experiment can be seen in Supplementary Tables 12–14 and Figure 4. For the parametric effects on context experiments, we observed significant BOLD response differences when contrasting the older with young adults (see Fig. 6, and Supplementary Table 12). These BOLD response differences were seen in the brain regions including the left inferior frontal gyrus (−48, 28, 20), left temporal anterior fusiform gyrus (−34, −2, −40), posterior left temporal fusiform cortex (−38, −28, −32), and superior parietal lobe (8, −62, −66), indicating stronger parametric BOLD response in these brain regions of older adults associated with increased mappings of context experiment across blocks. For the parametric effects on dimension experiments, we observed significant BOLD response differences when contrasting the older with young adults (see Supplementary Table 13). These BOLD response differences were seen in the brain regions including the right inferior parietal lobule (44, −66, 24), left middle temporal gyrus (−38, −60, 12), left superior parietal lobule (−14, −56, 68), and left frontal orbital cortex (−42, 24, −18), indicating stronger parametric BOLD response in these brain regions of older adults associated with increased mappings of dimension experiment across blocks. For the parametric effects on feature experiments, we observed significant BOLD response differences when contrasting the older with young adults (see Supplementary Table 14). These BOLD response differences were seen in the brain regions including middle/superior frontal gyrus (30, −6, 64), right precentral gyrus/premotor cortex (32, −14, 64), posterior temporal fusiform cortex (−32, −40, −22), and juxtapositional lobule cortex (formerly known as the supplementary motor cortex) (0, −12, 54), indicating stronger parametric BOLD response in these brain regions of older adults associated with increased mappings of feature experiment across blocks. No significant difference in parametric brain activation when contrast with the older adults to young adults during the response experiment was observed. Also, contrasting young adults with older adults observed no significant differences across experiments. Specifically, blocked parametric effects obtained in the group-level voxel-based contrasts between groups were subsequent with ROI analyses. Group functional ROI analysis was used to verify the characteristic event-related signal change in voxels identified by a prior block-level contrast. ROI results confirm these regions’ significant differences between the young and older adults. Specifically, older adults exhibit stronger BOLD response in left inferior frontal gyrus (−48, 28, 20; *t* = 2.907; df = 36; *P* = 0.0006), left temporal anterior fusiform gyrus (−34, −2, −40; *t* = 2.228; df = 36; *P* = 0.0032), posterior left temporal fusiform cortex (−38, −28, −32; *t* = 4.317; df = 36; *P* = 1.184e−4), and superior parietal lobe (8, −62, −66 (*t* = 3.8; df = 36; *P* = 5.379e−4); 4, −72, 52 (*t* = 5.294; df = 36; *P* = 6.106e−6)) across the parametric context competition. For the parametric dimension competition, older adults exhibit stronger BOLD response in right inferior parietal lobule (44, −66, 24; *t* = 2.547; df = 36; *P* = 0.015), left middle temporal gyrus (−38, −60, −12; *t* = 2.934; df = 36; *P* = 0.006), left superior parietal lobule (−14, −56, 68; *t* = 3.052; df = 36; *P* = 0.004), and left frontal orbital cortex (−42, 24, −18; *t* = 2.263; df = 36; *P* = 0.03). For the parametric feature competition, older adults exhibit stronger BOLD response in right precentral gyrus (30, −6, 64; *t* = 3.19; df = 36; *P* = 0.003), right premotor cortex (32, −16, 68; *t* = 3.47; df = 36; *P* = 0.001), left temporal fusiform cortex (−32, −40, −22; *t* = 3.656; df = 36; *P* = 8.125e−4), right premotor cortex (32, −20, 56; *t* = 2.398; df = 36; *P* = 0.022), and juxtapositional lobule cortex (formerly known Supplementary Motor Cortex) (0, −12, 54, *t* = 2.780; df = 36; *P* = 0.009). Moreover, ROI analysis based on prior findings with an identical task (Badre and D’Esposito 2007) was also analyzed. In their findings, a rostro–caudal gradient was evident as the level of abstraction increased. Specifically, response competition activated the left dorsal premotor cortex (PMd; −30 −10 68), and ROI analysis reveals similar sensitivity of dorsal premotor cortex (PMd) activation to the parametric response competition experiments (see Fig. 6*A*). Other regions from prior findings (Badre and D’Esposito 2007) also observed similar sensitivity of brain activation (see Fig. 6) in pre-PMd = left anterior dorsal premotor cortex(−38, 10, 34), IFS = left inferior frontal sulcus (−50, 26, 24), and FPC = left frontopolar cortex (−36, 50, 6). Moreover, the seed-based correlation between age differences in brain activations and behavioral performances (i.e., mRT difference and working memory) was reported in Tables 2 and 3.

**Table 2:** Seed-based correlation between parametric effect on age differences of ROIs and mean changes in mRT difference for each experiment

Experiment	Brain area	MNI coordinates			Pearson’s r	*P*	*BF_10_*
		*x*	*y*	*z*			
Context	Left inferior frontal gyrus	−48	28	−20	−0.642	1.398e−5^*^^*^^*^	1771.274^*^^*^^*^
	Left temporal anterior fusiform gyrus	−34	−2	−40	−0.340	0.037^*^	11.652^*^
	Left temporal fusiform cortex, posterior division	−38	−28	−32	−0.566	2.132e−4^*^^*^^*^	148.381^*^^*^^*^
	Superior parietal lobe	8	−62	66	−0.525	7.225e−4^*^^*^^*^	49.567^*^^*^
	Superior parietal lobe	4	−72	52	−0.613	4.211e−5^*^^*^^*^	646.613^*^^*^^*^
Dimension	Right inferior parietal lobule	44	−66	24	−0.269	0.102	0.731
	Left middle temporal gyrus, temporo-occipital part	−38	−60	12	−0.207	0.212	0.426
	Left superior parietal lobule	−14	−56	68	−0.030	0.858	0.205
	Left frontal orbital cortex	−42	24	−18	−0.127	0.449	0.266
Feature	Right precentral gyrus/premotor cortex	30	−6	64	−0.253	0.125	0.627
	Right precentral gyrus/premotor cortex	32	−14	64	−0.217	0.190	0.461
	Right precentral gyrus/premotor cortex	32	−16	68	−0.176	0.289	0.347
	Temporal fusiform cortex, posterior division	−32	−40	−22	−0.142	0.395	0.286
	Right precentral gyrus/premotor cortex	32	−20	56	−0.252	0.127	0.620
	Juxtapositional lobule cortex (formerly known as supplementary motor cortex)	0	−12	54	−0.031	0.853	0.205

^*^ *P* < 0.05;

^*^^*^^*^ *P* < 0.001; Behavioral performance (i.e., mRT difference scores (dRT)) was calculated by subtracting the average mRTs from the RT of correct trials across participants in each experiment.

**Table 3:** Seed-based correlation between parametric effect on age differences of ROIs and working memory capacity for each experiment across participants

Experiment	Brain area	MNI coordinates			Pearson’s r	*P*	*BF_10_*
		*x*	*y*	*z*			
Context	Left inferior frontal gyrus	−48	28	−20	−0.241	0.145	0.563
	Left temporal anterior fusiform gyrus	−34	−2	−40	−0.510	0.001^*^^*^	34.900^*^^*^
	Left temporal fusiform cortex, posterior division	−38	−28	−32	−0.457	0.004^*^^*^	10.999^*^
	Superior parietal lobe	8	−62	66	−0.417	0.009^*^^*^	5.337
	Superior parietal lobe	4	−72	52	−0.464	0.003^*^	12.680^*^
Dimension	Right inferior parietal lobule	44	−66	24	−0.322	0.049^*^	1.316
	Left middle temporal gyrus, Temporo-occipital part	−38	−60	12	−0.354	0.029^*^	2.739
	Left superior parietal lobule	−14	−56	68	−0.495	0.002^*^^*^	24.507^*^
	Left frontal orbital cortex	−42	24	−18	−0.350	0.031^*^	1.895
Feature	Right precentral gyrus/premotor cortex	30	−6	64	−0.204	0.219	0.419
	Right precentral gyrus/premotor cortex	32	−14	64	−0.308	0.030^*^	1.115
	Right precentral gyrus/premotor cortex	32	−16	68	−0.261	0.113	0.678
	Temporal fusiform cortex, posterior division	−32	−40	−22	−0.472	0.003^*^^*^	14.970^*^
	Right precentral gyrus/premotor cortex	32	−20	56	−0.388	0.035^*^	3.303
	Juxtapositional lobule cortex (formerly known as supplementary motor cortex)	0	−12	54	−0.407	0.018^*^	4.494

^*^ *P* < 0.05; ^*^^*^^*^P < 0.001; Behavioral performance (i.e., mRT difference scores (dRT)) was calculated by subtracting the mean RTs from the RT of correct trials across participants in each experiment.

### Mediation Analysis

In the mediation model, our goal is to determine whether the age-related functional changes are the reflection of atrophy. The model parameters are as follows: *X* is the age, *Y* is the brain regions of age differences in task-related functional changes from each experiment, and *M* is the gray matter volumes across all participants. Of the experiments, we found significant indirect effects of gray matter volumes on three age differences in functional changes in context experiments (Supplementary Fig. 5). Specifically, significant mediation (indirect) effects of gray matter volumes were found in age differences of the superior parietal lobe (*z* = 4.126, *P* = 3.684e−5 [95% CI = 0.392 ~ 1.102]). The parameter estimates of mediation models were reported in the Supplementary Materials.

## Discussion

We adopted Badre’s fMRI paradigm (Badre and D’Esposito 2007) to investigate whether an age effect on frontal lobe activation, which is organized hierarchically based on abstract levels of representation, could be found. Consistent with our hypothesis on behavioral performance, increasing competition under each condition was found, which was associated with a parametric increase in mRT in both young and older groups although older adults showed additional general RT slowing. The efficiency score was also investigated to examine whether speed–accuracy tradeoffs in RT experiments with respect to the abstractness of the representation represent a strategic choice in the older group. Results of behavioral performances at different abstraction levels suggest both response time and IES score decline when the levels of abstraction increased. Specifically, age effects on behavioral performance can prolong the RT and efficiency in comparison with young adults, indicating poor performance accuracy in the older adults. Behavioral findings indicated that age-related slowing is seen in response speed in tasks that required the selection of sets of stimuli–response mappings. To address our first goal, the overall behavioral performance of young adults was consistent with the original study by Badre, while older adults exhibited declines in task performances more than young adults. Furthermore, the behavioral performances across four experiments showed that working memory capacity appears to be negatively associated with RT and positively associated with efficiency score, indicating age-related differences in working memory capacity influence the processing levels of abstractions.

On the fMRI findings, BOLD results demonstrated a clear trend in hierarchical gradient patterns depending on abstraction levels in both age groups, while older adults exhibited overaction in frontal-associated regions. Specifically, our results demonstrate that coherent patterns of fMRI activation when compared with a prior study (Badre and D’Esposito 2007) in which the functional gradient along the rostro–caudal axis of the frontal lobe exists whereby progressively anterior subregions of the frontal lobe are associated with higher-order processing requirements of more abstract action planning. ROI results from group activation support these findings. Importantly, age-related differences of task-evoked brain activation patterns were variable within the four different difficulty manipulations, reflecting more hierarchical activation across different task manipulations. These findings provide empirical support for the hypothesis that cognitive control is organized in a representational hierarchy along the rostro–caudal axis of the frontal lobes (Badre 2008; Badre et al. 2009; Badre and D’Esposito 2007, 2009). Moreover, our findings are also in agreement with a recent view in which the functional gradient at each order of cognitive control is not restricted to the frontal lobe but also includes regions of medial frontal, parietal, and temporal cortices (Choi et al. 2018). This observation is consistent with a recent study in which it was reported that older adults were recruiting more distributed cortical resources as task demands increased (Crowell et al. 2020). Furthermore, age differences in overactivation in different brain regions, including inferior frontal gyrus, frontal orbital cortex, temporal fusiform gyrus, superior parietal lobe, precentral gyrus, premotor cortex, and supplementary motor cortex, in three of four experiments were observed. These age-related functional gradient differences also were noted along the rostro–caudal axis of the frontal lobe depending on the levels of abstractions. General findings were consistent with numerous neuroimaging studies (Li et al. 2015; Fjell et al. 2017; Escrichs et al. 2021) that demonstrated that older adults tend to activate the brain to a greater extent than younger adults during the performance of a task (Spreng et al. 2010). This observation is commonly found to be activated in frontal regions (Davis et al. 2008; Park and Reuter-Lorenz 2009; Reuter-Lorenz and Park 2014). This finding is typically interpreted as evidence for neurocognitive compensation (Reuter-Lorenz and Cappell 2008) coupled with behavioral performance if a significant, positive, brain–behavior relationship is observed. In our study, we observed overrecruitment activation primarily in the context experiment, which is based on the information from a current temporal frame (e.g., episodic control). Importantly, only age differences in the context experiment showed a significant negative correlation between parametric brain activity and dRT performance across mappings, indicating older adults possibly attempt to recruit overactive more cortical activity to delay cognitive decline while at the highest level of task-difficulty manipulation. According to the frontal lobe hypothesis (Cabeza and Dennis 2012), the observation of attempted compensation would be greatest in those that need it the most but decline with advanced brain deterioration. With respect to our hypothesis, a negative correlation between activation and task performance would suggest that such activation is detrimental, rather than compensatory, to task performance (Qin and Basak 2020). Specifically, observed negative correlations between age difference-related brain activity in context experiment and dRT were found (see Table 2), indicating overrecruitment of brain activity in these regions associated with the relatively poor individual performance of RT deviation compared with the average performance.

**Figure 5:**
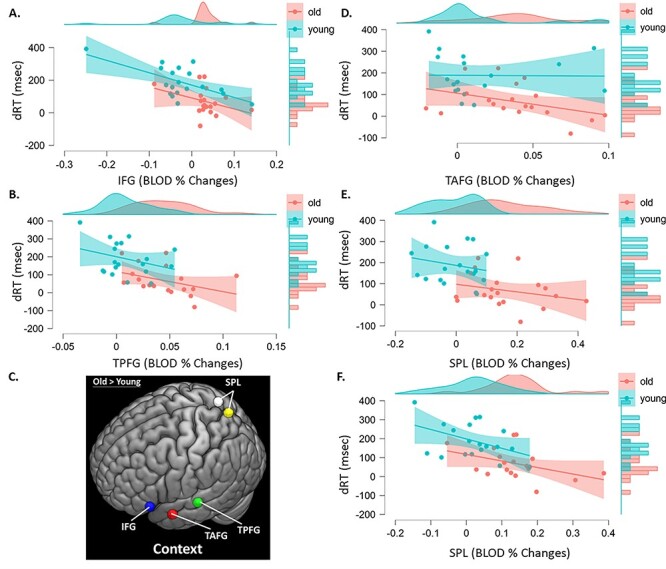
Scatter plots between age difference of ROIs and dRT on parametric effects of context experiment across participants. Correlation between age difference in brain activity and dRT of context experiment across participants. dRT = delta reaction time; msec = milliseconds; old = older adults; young = young adults; IFG = inferior frontal gyrus; TAFG = temporal anterior fusiform gyrus; TPFG = temporal posterior fusiform gyrus; SPL = superior parietal lobule.

To verify these patterns, the results reported in the scatter plot (see Fig. 5) show that the trends of correlation in both groups reveal that stronger overrecruitment brain activity in older adults covaries negatively with task performances with high cognitive control demands. For results illustrated in Figure 5*D* as an example, the task performance of young adults covaried less with overrecruitment of brain activity in the temporal anterior fusiform gyrus, while older adults exhibited stronger brain activity in this same region to involve in their task performance. With regards to the PASA hypothesis, our primary observed overactivation in the context experiments for older adults showed no reduction in activity in occipital regions (such as the visual processing area) in addition to worse performance (see Supplementary Table 15). According to the PASA hypothesis, older adults with the weakest occipital recruitment are the ones who are more dependent on PFC overrecruitment to maintain performance. In a classic study (Grady et al. 1994), older and younger adults were matched in terms of accuracy but differed in RTs, so the authors further suggested that additional recruitment of PFC functions would allow older adults to maintain a good accuracy level at the expense of slower RTs. However, our current results did not seem to support such a compensatory theory. Also, for the HAROLD model, age differences of compensatory activity showed no positive correlation task performance despite the observed patterns of bilaterality of PFC activation in older adults during the highest task demands. For example, Reuter-Lorenz et al. (2000) found that older adults who showed bilateral recruitment during working memory tasks showed faster reaction times (Reuter-Lorenz et al. 2000). Likewise in a recent study by Bergerbest et al. (2009), activation in the contralateral PFC region recruited by older adults in the right PFC was positively correlated with the magnitude of behavioral priming in these people. Nonetheless, possible mechanisms, including a lack of efficiency in the utilization of neural resources or a reduction in the selectivity of responses, known as dedifferentiation (Grady 2008, 2012), may also explain age-related increases in brain activity. Age-related overrecruitment has been interpreted in a way of neural dedifferentiation, especially when it is observed to covary negatively with task performance (i.e., deterioration) (Stevens et al. 2008; De Chastelaine et al. 2011). This observation was consistent with the finding from most aging neuroimaging studies in which older adults activate extra brain regions compared with the regions that are activated in younger adults during a cognitive task (Spreng et al. 2010). Such overactivation in older adults has been reported during tasks of episodic memory retrieval (Cabeza 2002; De Chastelaine et al. 2011). According to our design of the task, some of these observed overactivations in older adults on context experiments may reflect age-related differences in neural recruitment for the most control-related demands.

**Figure 6:**
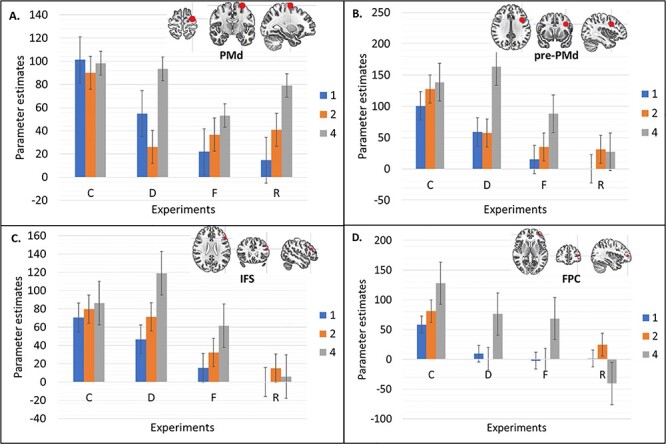
BOLD responses of parametric effect across the four experiments based on ROIs from Badre and D’ Esposito (2007). A rostro–caudal gradient was evident as the level of abstraction increased. Specifically, BOLD response of response competition activated in PMd (*A*), BOLD response of feature competition activated in the pre-PMd (*B*), BOLD response of dimension competition activated the IFS (*C*), and context competition associated with BOLD response activated the FPC (*D*). PMd = left dorsal premotor cortex(−30, −10, 68); pre-PMd = left anterior dorsal premotor cortex(−38, 10, 34); IFS = left inferior frontal sulcus (−50, 26, 24); FPC = left frontopolar cortex (−36, 50, 6).

These age differences of brain activations between the parametric effect across mappings obtained in each experiment, showing overactivation of brain regions in older adults, reflect functional mechanisms either in compensatory activity or in dedifferentiation. Overactivation in prefrontal brain areas has been previously observed in older adults during fMRI tasks, giving rise to different theories (Davis et al. 2008; Reuter-Lorenz and Cappell 2008). One of which is the compensation-related utilization of the neural circuits hypothesis (Reuter-Lorenz and Cappell 2008). According to this utilization of the neural circuit’s hypothesis, cognitive demands of the task play a critical role in age-related differences in neural recruitment. For lower task demands, older adults are hypothesized to recruit additional brain regions to compensate for age-related neural degradation, resulting in similar performance levels to those of younger adults. However, the dedifferentiation hypothesis does not necessarily predict that age-related overactivation reflects functional compensation (Rajah and D’Esposito 2005). This hypothesis considers that with increasing age, brain regions become less specialized in their functions, resulting in generalized spreading of brain activity to other brain regions. For example, younger adults were found to recruit the medial temporal lobe for explicit learning and the striatum for implicit learning, whereas older adults did not show a clear neural distinction between these two types of memory (Cabeza and Dennis 2012). Similarly, category-specific brain regions, which respond selectively to one type of item or task (such as the fusiform region for faces) in younger adults, become less selective in older adults (Park et al. 2012; Abdulrahman et al. 2017). A variation in the dedifferentiation hypothesis considers such overactivation to be due to a reduced ability to suppress task-irrelevant brain activity by older adults, which impairs their optimal engagement of task-relevant regions (Logan et al. 2002). Along this line, if maladaptive overactivation is observed in older adults, activation of such task-irrelevant regions may add detrimental noise to the memory system and contribute to worsened updating (Qin and Basak 2020). Previous studies have shown that older adults exhibit deficient working memory performance that is related to a selective deficit in inhibiting sensory processing activity to irrelevant stimuli (Gazzaley et al. 2005; Chadick et al. 2014). Importantly, such decline in suppressing irrelevant stimulus processing is associated with decreased activity in the frontal lobe in addition to decreased functional connectivity between the sensory cortex and the frontal lobe (Chadick et al. 2014). Recent studies on age-related overrecruitment in the frontal lobe depending on memory load may be attributed to limited-resource functional compensatory mechanisms (Cappell et al. 2010; Qin and Basak 2020). Accordingly, older adults may exhibit deficient working memory performance due to overloading limited memory stores with irrelevant information. Parallel to our findings of working memory, a correlation between working memory performance and overactivation brain regions showed a strong and consistent negative association in most of the brain regions with overactivations (Table 3), especially in parietal regions (Jonides et al. 1998), indicating overactivation could be attributed to a reduced ability to suppress task-irrelevant brain activity by older adults, which impairs their optimal engagement of task-relevant regions (Crone et al. 2006; Mattay et al. 2006). Moreover, the correlation between task performance in each mapping and working memory capacity across participants also showed a consistent link between performance efficiency (such as IES score) and working memory capacity (Supplementary Table 16), suggesting a critical role for working memory in the process of levels of task difficulty. Hence, increased frontal-associated activity may be more related to reduced neural specificity than to compensation at the lower level of control demand. This process may explain why our findings did not observe associations between the age difference of the parametric brain activity for other experiments (e.g., dimension, feature, and response) and dRT performances.

 Furthermore, we observed no age differences in the parametric effects of the response experiment despite a decrease in behavioral performance, suggesting lower control demands in sensorimotor representation did not differ in brain activity recruitment. A possible explanation would be either young adults have increased brain activity when task difficulty increased or older adults failed to show any significant neuromodulation in response to motor task demands. Nonetheless, age differences in parametric effects of feature experiments become more prominent in the motor cortex for stimulus–response mappings, suggesting functional dysregulation of motor cortex excitability during sensorimotor control processing with this deficit becoming progressively evident with greater task complexity. Other than the predicted activation observed along the posterior to anterior frontal lobe gradient, the parametric analyses with ROI also confirmed the brain activation in regions of primary motor, parietal, and inferior temporal cortices across the experiment. Likewise, these regions also reflected the age difference in parametric effects on brain activity, showing overrecruitment in older adults. ROI analysis further showed brain activation in these regions across experiments in response to control demands (competitions). The observation of these brain regions suggests a general sensitivity to the increase in control demands without being selective to the type of competition at a particular representational level. These regions also reflect the age difference of parametric effects across experiments, meaning these brain regions may serve as shared functional connectivity to process levels of abstraction and control demands. Activation of these regions, which are not positively correlated with task performance, has been interpreted as a reduced specificity in neural recruitment during tasks in older adults (Koen and Rugg 2019). It is plausible that for young adults either a difference in neuromodulation with respect to executive control or overactivation that is compensatory to task performance occurs, whereas older adults may fail to modulate cognitive control and show maladaptive, reduced specificity in neural recruitment during tasks. However, if this overactivation is associated with a decrease in task performance, it can then be interpreted as maladaptive overactivation (Nashiro et al. 2018).

### Limitations of the Study and Future Directions

Before closing, some issues are worth mentioning, especially for the applications in age-related neuroimaging studies (Luna et al. 2010). Maturation and aging are important life periods that are linked to drastic brain reorganization processes (Rakic et al. 1994; Petanjek et al. 2011). Brain reorganization processes throughout a person’s lifetime demonstrate brain circuit remodeling at the structural and functional levels that are parallelized by physiological trajectories during maturation and healthy aging. In this study, the developmental stages of brain maturation in the young adult group may affect the neurobiological aging profiles (Petanjek et al. 2019). These developmental processes within young adults could underlie a better integration of structural and functional communication between brain regions with age. Future studies should consider the developmental stages in young and older groups that may weaken the sample representative. Nevertheless, in this study, the young age group was going through the final stage of cerebral cortex development, which paradoxically made the young group an ideal control group in which the comparison between the two age groups might reflect the growing maturity versus the aging decline.

Moreover, there is substantial individual variability in the aging process between men and women. For example, sex hormones organize structural connections and activate the brain areas they connect (Gong et al. 2011; Arain et al. 2013). Therefore, when examining brain development and aging or when investigating the possible biological mechanisms of developmental changes, the contribution of gender should not be ignored (Gong et al. 2011; Arain et al. 2013). Future studies should consider the sex differences of brain maturity when investigating the age differences of brain organization.

In this study, two of the most challenging mappings (iC4 and D4) failed to show slower RT for older adults; a possible explanation for this finding may be due to the ceiling effect of RT for both older and young adults. This inference can be supported by parametric effects of context experiments in those older adults who show more extensive frontal lobe activation than do young adults across different mappings. One possible explanation is that age-related overactivation is driven by a combination of control demand–related overactivation of task-relevant regions in response to increasing cognitive control demands, and thus overrecruitment of additional brain regions by older adults that are not recruited by younger adults when performing the task (less functional specifications) occurs. While task difficulty is typically indexed by a proportional increase in RT as a function of increased difficulty in a given task (Basak and Verhaeghen 2011), control demand refers to our responses to the different types of cognitive challenges encountered within the same complex task. Therefore, each type of control demand (abstractness of representation) is typically calculated from trials of two different levels of task difficulty, by subtracting the average RTs of the less difficult trials from the average RTs of the more difficult trials. A complex task, such as a multitasking paradigm, may involve different types of control demands, which in turn could recruit both overlapping in addition to distinct cognitive control demands in brain regions.

Furthermore, the calculated power (1—β) for the statistical *t*-test (Erdfelder et al. 1996) was relatively low (0.32), indicating the probability of a type II error (Brydges 2019). Therefore, future studies should try to aim for recruiting more older adults to participate in task-related fMRI studies. Alternatively, other statistical power methods, such as BF, can also be applied to test the hypothesis by using Bayesian inference with the strength of evidence (Keysers et al. 2020). Despite the challenges posed to older adults for completing this task in the MRI scanner, this is the first study to investigate whether the rostral-to-caudal functional gradient of the frontal lobe is disrupted by age. Recent studies have shown the difficulties in recruiting older adults for aging research (Mody et al. 2008), a finding that may present concerns for the current study (Brydges 2019). Nonetheless, a recent well-cited meta-analysis review of seventy-seven studies (Spreng et al. 2010) on age differences in brain activity reports the age of young participants was on average 24.81 ± 2.8 years, while that of old participants was 68.81 ± 3.9 years across 77 studies. These age results were identical to the ages of both young and older adults in the current study. This finding also can be viewed in the aforementioned meta-analysis review of seventy-seven studies (Spreng et al. 2010) on task-related brain activity differences that the average number of participants in fMRI studies was 14.54 ± 6.1 years. Depending upon the size of the effect that one wishes to detect, the small sample size may be unwise, but a small sample size does not invalidate the test. Although challenges for recruiting older adults in aging research exist, we believe the current study would have a substantial contribution to better understand the age effect of the functional organization on the frontal lobe.

Additionally, one potential confounding effect from brain structural abnormalities may be undermined by the overrecruitment of brain activity in older adults as a strategy to compensate for individual variability of task performance (Venkatraman et al. 2010; Hinault et al. 2020). To mitigate the confounding effects, we performed an additional mediation analysis of gray matter volumes on the relationships between age differences of ROIs in context experiments and cognitive performance was conducted. Results suggest that possibilities of age differences in functional changes that can be affected by the individual atrophy levels, especially in the left temporal fusiform cortex, can exist. Nonetheless, the results indicate compensatory brain activity in the left temporal fusiform cortex may need to be interpreted with a warrant as to the presence of underlying brain connectivity structural abnormalities.

In this study, we applied Badre’s fMRI paradigm (Badre and D’Esposito 2007) to investigate the age differences of functional specialization and organization in the frontal lobe. Behavioral findings suggested a parametric effect of behavioral performance changes across experiments and mappings, overall slowing RT, and poor efficiency of selection of a set of stimulus–response mappings in older adults was observed. Results of behavioral performances at different abstraction levels suggest both response time and efficiency decline when the levels of abstraction increased. Task performances at different levels of abstract representations and age difference overactivations are associated with working memory, highlighting the key role of working memory in processing different levels of abstract representations and control demands. Together with fMRI findings, the current results suggest clear trends of specialization and organization along the rostro–caudal axis in both groups can be found, and further brain–behavior seed-based correlation suggesting a reduction of neural specificity for the highest level of control demands experiment. Less specialization when lower task control demands increased in the older adults was shown, resulting in generalized spreading of brain activity to other brain regions. Attempts to compensate mechanisms are activated when performing higher task control demands, resulting in overrecruitment of additional brain regions that are detrimental to task performance. Age differences in overactivation in brain regions were observed in three of four experiments, including inferior frontal gyrus, frontal orbital cortex, temporal fusiform gyrus, superior parietal lobe, precentral gyrus, premotor cortex, and supplementary motor cortex. ROI analysis reveals identical parametric activations as shown in a study by Badre and D’Esposito (2007) for fMRI across mappings in each experiment. Moreover, age differences in overactivation and general activation across experiments in the primary motor cortex, parietal lobule, and fusiform gyrus may serve as shared mechanisms underlying tasks that are required for the selection of sets of stimulus–response mappings. These findings provide empirical support for the hypothesis that cognitive control is organized in a representational hierarchy along the rostro–caudal axis of the frontal lobes in both older and young adult groups. Overall, findings suggest age differences in task-evoked brain overactivation changes, indicating the loss of neural specificity in task performances with higher control demand for older adults.

## Supplementary Material

rev1_Supplementary_Material_zf2_proof_corrected_bhab382

## Notes

We thank Mind Research and Imaging Center (MRIC) at National Cheng Kung University for consultation and instrument availability. MRIC is supported by the MOST. *Conflicts of Interest*: The authors have none to declare.
