# Supplementary material for: Age Differences of the Hierarchical Cognitive Control and the Frontal Rostro–Caudal Functional Brain Activation
Source: Cereb Cortex. 2021 Nov 2;32(13):2797–815. doi: 10.1093/cercor/bhab382 (PMC9247418; doi:10.1093/cercor/bhab382)
Supplement: rev1_Supplementary_Material_zf2_proof_corrected_bhab382 [file rev1_supplementary_material_zf2_proof_corrected_bhab382.docx]

**Supplementary Material**

Supplementary Fig 1. Task schematic paradigm for response experiment.


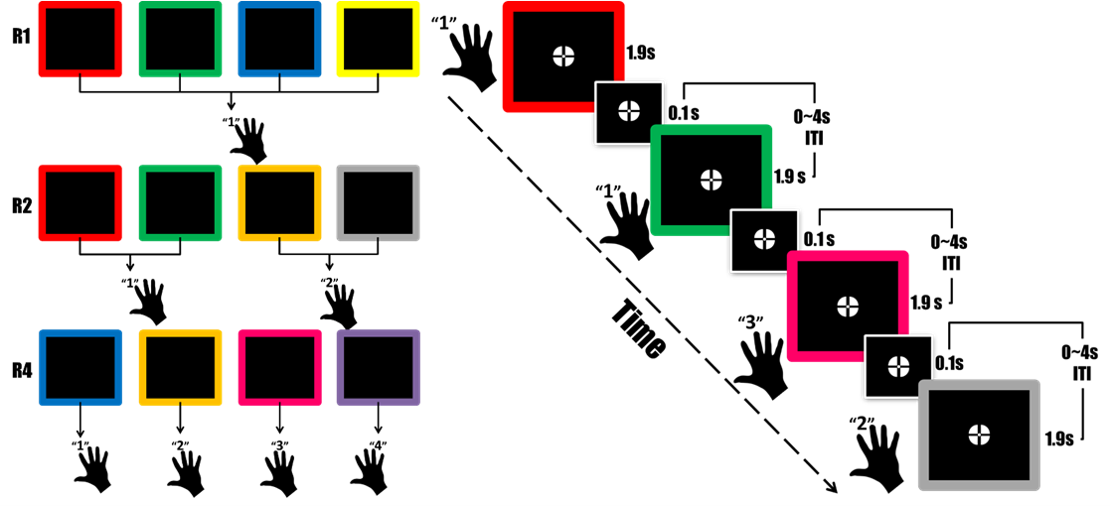


Supplementary Fig 2. Task schematic paradigm for feature experiment.


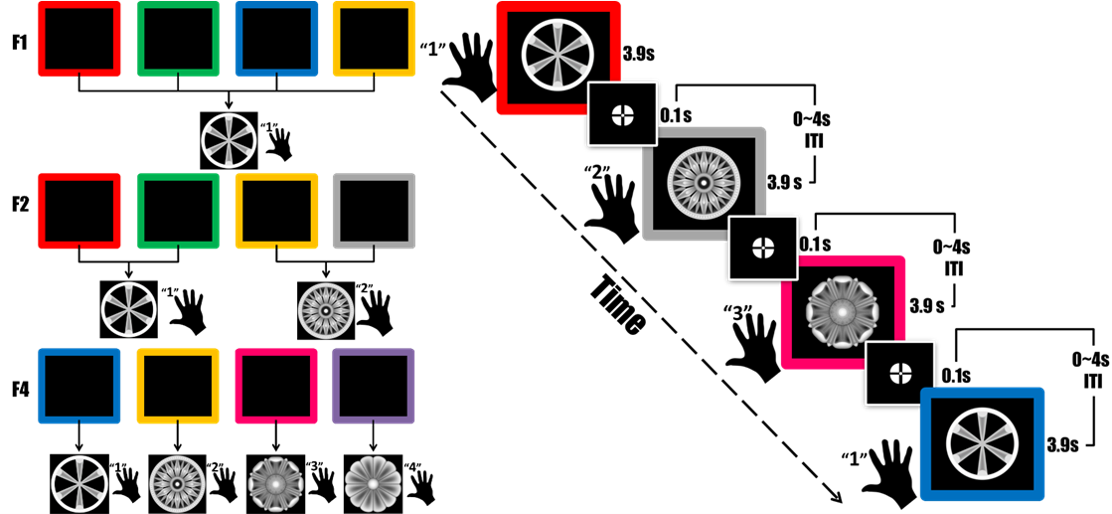


Supplementary Fig 3. Task schematic paradigm for dimension experiment.


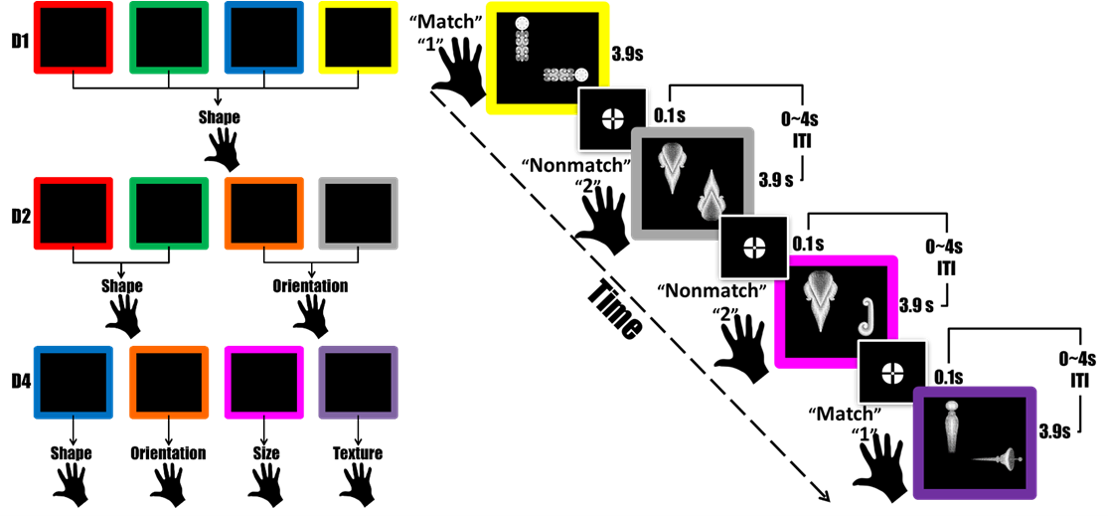


Supplementary Fig 4. Task schematic paradigm for context experiment.


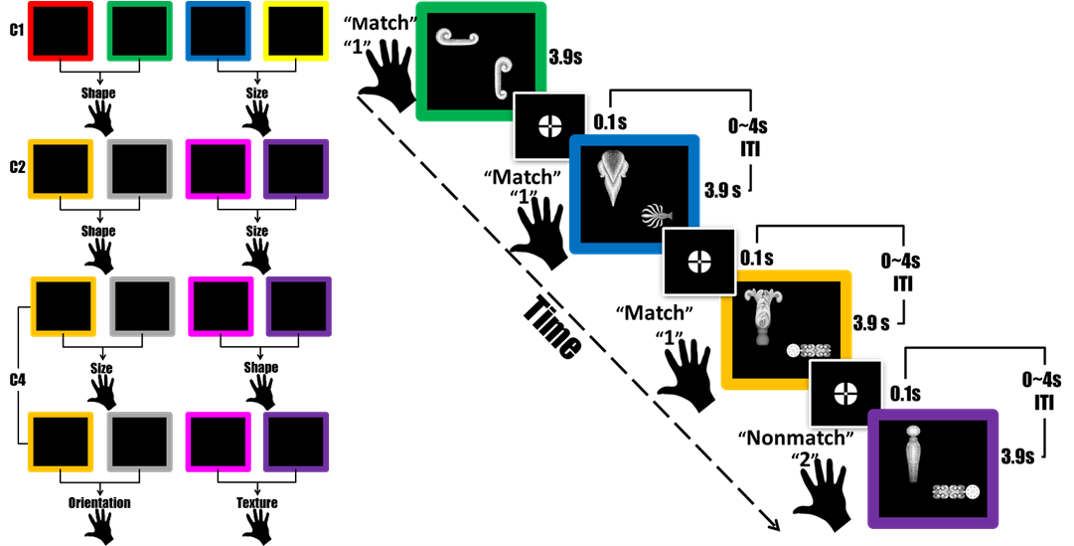


Supplementary Fig 5. Mediation model of age, grey matter volumes and age differences in functional changes of the left temporal fusiform cortex.


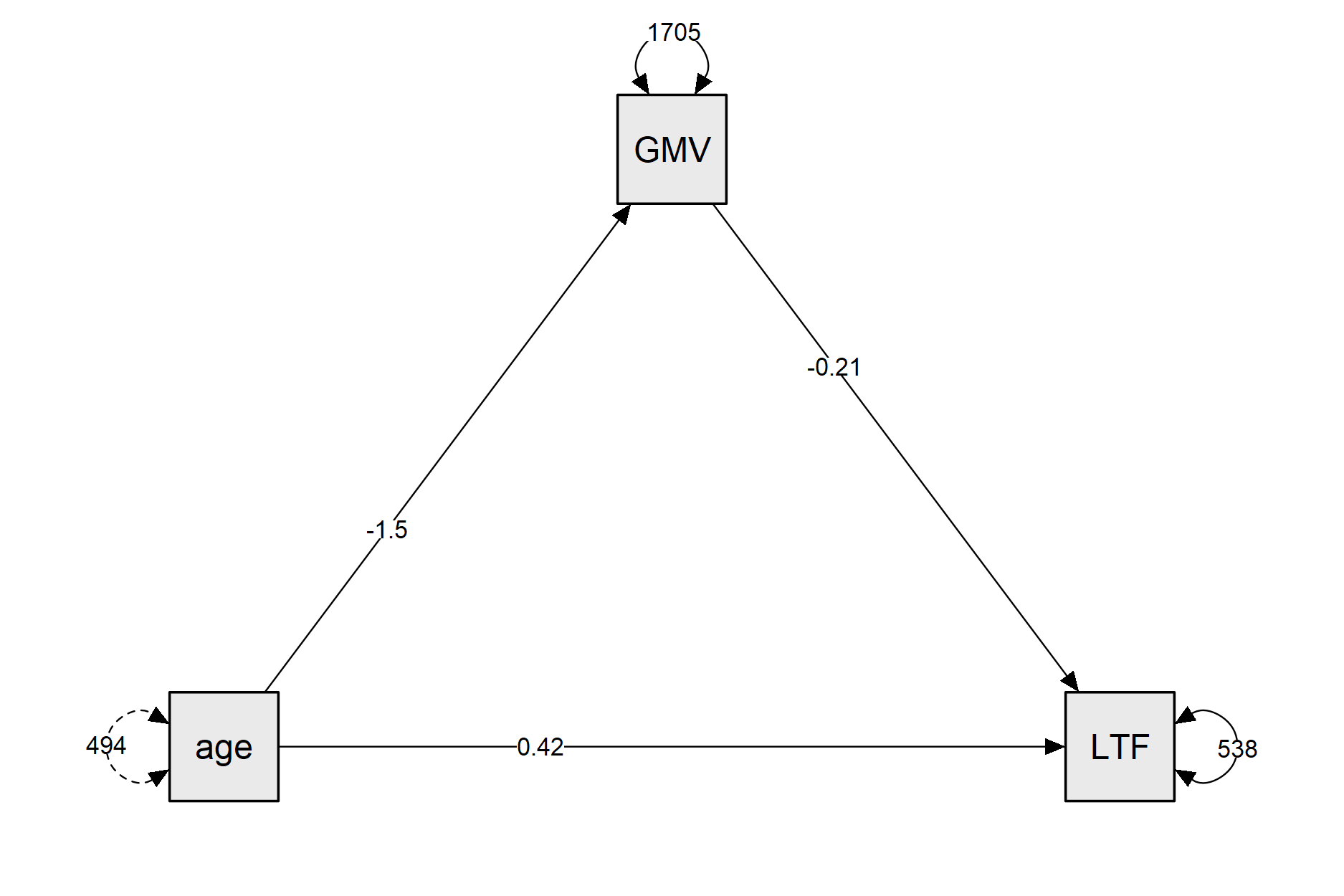


Supplementary Table 1. Demographic information about participants in the current study

| ID | Gender | Age | Education | BDI-II | WM | MoCA |
| --- | --- | --- | --- | --- | --- | --- |
| AF035 | male | 66 | 12 | 9 | 22 | 30 |
| AF044 | male | 79 | 20 | 1 | 25 | 28 |
| AF053 | female | 64 | 16 | 15 | 23 | 28 |
| AF054 | female | 61 | 16 | 3 | 22 | 30 |
| AF055 | male | 65 | 12 | 5 | 21 | 28 |
| AF056 | male | 63 | 16 | 5 | 28 | 29 |
| AF057 | male | 63 | 12 | 9 | 23 | 28 |
| AF058 | female | 62 | 16 | 2 | 21 | 30 |
| AF059 | male | 71 | 12 | 18 | 15 | 26 |
| AF060 | female | 64 | 16 | 3 | 21 | 29 |
| AF061 | male | 78 | 18 | 0 | 15 | 30 |
| AF062 | male | 62 | 16 | 2 | 23 | 26 |
| AF063 | female | 62 | 12 | 0 | 26 | 30 |
| AF064 | female | 70 | 12 | 1 | 26 | 30 |
| AF065 | male | 74 | 12 | 0 | 19 | 28 |
| AF066 | male | 73 | 16 | 9 | 20 | 29 |
| AF067 | male | 70 | 16 | 1 | 26 | 29 |
| AF070 | female | 60 | 16 | 1 | 25 | 30 |
| AF072 | male | 74 | 12 | 6 | 21 | 29 |
| AF013 | male | 20 | 15 | 14 | 27 | 30 |
| AF019 | male | 22 | 17 | 29 | 28 | 29 |
| AF020 | female | 20 | 15 | 20 | 28 | 29 |
| AF029 | male | 39 | 18 | 0 | 28 | 29 |
| AF030 | female | 37 | 20 | 4 | 29 | 30 |
| AF031 | female | 21 | 15 | 3 | 27 | 30 |
| AF033 | male | 24 | 17 | 4 | 25 | 27 |
| AF034 | female | 20 | 15 | 4 | 29 | 30 |
| AF037 | female | 26 | 18 | 10 | 30 | 30 |
| AF038 | male | 24 | 18 | 1 | 29 | 28 |
| AF039 | female | 23 | 14 | 22 | 28 | 30 |
| AF040 | female | 22 | 17 | 5 | 27 | 29 |
| AF041 | male | 21 | 14 | 6 | 24 | 30 |
| AF042 | female | 24 | 19 | 8 | 29 | 30 |
| AF043 | male | 27 | 19 | 0 | 27 | 29 |
| AF045 | male | 25 | 18 | 3 | 29 | 30 |
| AF050 | male | 24 | 18 | 10 | 28 | 30 |
| AF051 | male | 22 | 16 | 5 | 28 | 29 |
| AF071 | male | 21 | 16 | 9 | 27 | 30 |

*ID=identification code of participant, BDI-II=Beck Depression Inventory-II, WM=working memory span, MoCA= Montreal Cognitive Assessment for Dementia.

Supplementary Table 2. Behavioral results across all competitions

| Conditions | Group | N | Mean RT (msec) | SD | SE |
| --- | --- | --- | --- | --- | --- |
| C1 | elderly | 19 | 1533.317 | 316.664 | 72.648 |
|  | young | 19 | 1340.422 | 264.024 | 60.571 |
| C2 | elderly | 19 | 1351.537 | 318.184 | 72.996 |
|  | young | 19 | 1049.012 | 220.231 | 50.525 |
| C4 | elderly | 19 | 1544.595 | 336.397 | 77.175 |
|  | young | 19 | 1477.547 | 235.133 | 53.943 |
| D1 | elderly | 19 | 1090.825 | 186.036 | 42.680 |
|  | young | 19 | 841.951 | 187.136 | 42.932 |
| D2 | elderly | 19 | 1551.965 | 294.507 | 67.565 |
|  | young | 19 | 1257.000 | 262.987 | 60.333 |
| D4 | elderly | 19 | 1510.672 | 345.811 | 79.335 |
|  | young | 19 | 1480.829 | 311.034 | 71.356 |
| F1 | elderly | 19 | 783.100 | 123.159 | 28.255 |
|  | young | 19 | 649.704 | 174.016 | 39.922 |
| F2 | elderly | 19 | 1274.729 | 218.019 | 50.017 |
|  | young | 19 | 1050.964 | 196.595 | 45.102 |
| F4 | elderly | 19 | 1422.734 | 205.026 | 47.036 |
|  | young | 19 | 958.563 | 187.551 | 43.027 |
| R1 | elderly | 19 | 500.171 | 118.457 | 27.176 |
|  | young | 19 | 376.976 | 84.848 | 19.465 |
| R2 | elderly | 19 | 797.993 | 110.422 | 25.333 |
|  | young | 19 | 647.846 | 136.886 | 31.404 |
| R4 | elderly | 19 | 1037.931 | 163.640 | 37.542 |
|  | young | 19 | 740.367 | 180.591 | 41.430 |

C=context experiment; b=block; D=dimension experiment; F=feature experiment; R=response experiment. RT=average reaction time; msec= millisecond; SD= standard deviation; ACC=accuracy.

Supplementary Table 3. RT of Badre’s task performance between groups

| Reaction Time | | | |
| --- | --- | --- | --- |
|  | t | df | p |
| C1 | 2.039 | 36.000 | 0.049 |
| C2 | 3.408 | 36.000 | 0.002 |
| C4 | 0.712 | 36.000 | 0.481 |
| D1 | 4.111 | 36.000 | 2.177e -4 |
| D2 | 3.256 | 36.000 | 0.002 |
| D4 | 0.280 | 36.000 | 0.781 |
| F1 | 2.727 | 36.000 | 0.010 |
| F2 | 3.322 | 36.000 | 0.002 |
| F4 | 7.281 | 36.000 | 1.408e -8 |
| R1 | 3.685 | 36.000 | 7.467e -4 |
| R2 | 3.721 | 36.000 | 6.740e -4 |
| R4 | 5.322 | 36.000 | 5.595e -6 |

C=context experiment; D=dimension experiment; F=feature experiment; R=response experiment, df=degree of freedom, p= p-value

Supplementary Table 4. IES of task performance between groups

| Efficiency (IES scores) | | | |
| --- | --- | --- | --- |
|  | t | df | p |
| C1 | 3.164 | 36.000 | 0.003 |
| C2 | 4.149 | 36.000 | 1.945e -4 |
| C4 | 2.364 | 36.000 | 0.024 |
| D1 | 4.509 | 36.000 | 6.662e -5 |
| D2 | 4.129 | 36.000 | 2.063e -4 |
| D4 | -0.626 | 36.000 | 0.535 |
| F1 | 2.855 | 36.000 | 0.007 |
| F2 | 4.550 | 36.000 | 5.891e -5 |
| F4 | 4.404 | 36.000 | 9.121e -5 |
| R1 | 3.465 | 36.000 | 0.001 |
| R2 | 2.865 | 36.000 | 0.007 |
| R4 | 2.805 | 36.000 | 0.008 |

C=context experiment; D=dimension experiment; F=feature experiment; R=response experiment, df=degree of freedom, p= p-value

Supplementary Table 5. Correlation coefficients of IES scores of fMRI task and behavioral demographic information

|  | | age | | edu | BDI-II | | MoCA | | FAB | WM | | C1 | | c2 | | C4 | | D1 | | D2 | | D4 | | F1 | | F2 | | F4 | | R1 | | R2 | | R4 | |
| --- | --- | --- | --- | --- | --- | --- | --- | --- | --- | --- | --- | --- | --- | --- | --- | --- | --- | --- | --- | --- | --- | --- | --- | --- | --- | --- | --- | --- | --- | --- | --- | --- | --- | --- | --- |
| age | — | |  | | |  | |  |  | |  | |  | |  | |  | |  | |  | |  | |  | |  | |  | |  | |  | |  |
| edu | -0.363* | | — | | |  | |  |  | |  | |  | |  | |  | |  | |  | |  | |  | |  | |  | |  | |  | |  |
| BDI-II | -0.308 | | -0.144 | | | — | |  |  | |  | |  | |  | |  | |  | |  | |  | |  | |  | |  | |  | |  | |  |
| MoCA | -0.301 | | 0.133 | | | -0.057 | | — |  | |  | |  | |  | |  | |  | |  | |  | |  | |  | |  | |  | |  | |  |
| FAB | -0.503** | | 0.369* | | | 0.114 | | 0.256 | — | |  | |  | |  | |  | |  | |  | |  | |  | |  | |  | |  | |  | |  |
| WM | -0.742*** | | 0.438** | | | 0.093 | | 0.381* | 0.387* | | — | |  | |  | |  | |  | |  | |  | |  | |  | |  | |  | |  | |  |
| C1 | 0.459** | | -0.162 | | | -0.049 | | -0.091 | -0.302 | | -0.31 | | — | |  | |  | |  | |  | |  | |  | |  | |  | |  | |  | |  |
| C2 | 0.594*** | | -0.099 | | | -0.066 | | -0.199 | -0.257 | | -0.604*** | | 0.552*** | | — | |  | |  | |  | |  | |  | |  | |  | |  | |  | |  |
| C4 | 0.303 | | -0.086 | | | -0.065 | | 0.139 | -0.307 | | -0.163 | | 0.541*** | | 0.455** | | — | |  | |  | |  | |  | |  | |  | |  | |  | |  |
| D1 | 0.62*** | | -0.083 | | | -0.142 | | -0.089 | -0.287 | | -0.46** | | 0.589*** | | 0.827*** | | 0.671*** | | — | |  | |  | |  | |  | |  | |  | |  | |  |
| D2 | 0.517*** | | -0.193 | | | -0.129 | | 0.066 | -0.159 | | -0.502** | | 0.565*** | | 0.481** | | 0.43** | | 0.605*** | | — | |  | |  | |  | |  | |  | |  | |  |
| D4 | -0.051 | | 0.232 | | | -0.283 | | 0.065 | 0.075 | | 0.104 | | 0.289 | | 0.087 | | 0.177 | | 0.24 | | 0.247 | | — | |  | |  | |  | |  | |  | |  |
| F1 | 0.483** | | -0.164 | | | -0.114 | | -0.187 | -0.299 | | -0.269 | | 0.501** | | 0.503** | | 0.335* | | 0.628*** | | 0.434** | | 0.281 | | — | |  | |  | |  | |  | |  |
| F2 | 0.585*** | | -0.227 | | | -0.229 | | -0.113 | -0.371* | | -0.307 | | 0.288 | | 0.513*** | | 0.41* | | 0.577*** | | 0.332* | | 0.135 | | 0.478** | | — | |  | |  | |  | |  |
| F4 | 0.654*** | | -0.448** | | | -0.093 | | -0.312 | -0.511** | | -0.611*** | | 0.235 | | 0.39* | | 0.16 | | 0.441** | | 0.228 | | -0.217 | | 0.33* | | 0.346* | | — | |  | |  | |  |
| R1 | 0.54*** | | -0.283 | | | -0.013 | | -0.139 | -0.208 | | -0.352* | | 0.352* | | 0.44** | | 0.308 | | 0.573*** | | 0.249 | | -0.086 | | 0.65*** | | 0.511** | | 0.622*** | | — | |  | |  |
| R2 | 0.415** | | -0.351* | | | -0.055 | | 0.019 | -0.001 | | -0.355* | | 0.169 | | 0.245 | | 0.149 | | 0.362* | | 0.322* | | 0.004 | | 0.417** | | 0.405* | | 0.257 | | 0.592*** | | — | |  |
| R4 | 0.464** | | -0.419** | | | 5.567e -4 | | -0.164 | -0.311 | | -0.414** | | 0.156 | | 0.196 | | 0.096 | | 0.303 | | 0.182 | | -0.042 | | 0.371* | | 0.362* | | 0.653*** | | 0.716*** | | 0.444** | | — |

* p < .05, ** p < .01, *** p < .001

Supplementary Table 6. Mediation (indirect) effects of grey matter volume on age differences in brain functional changes of context experiment.

| Indirect effects | | | | | | | | | | | | | | | | | | | | | |
| --- | --- | --- | --- | --- | --- | --- | --- | --- | --- | --- | --- | --- | --- | --- | --- | --- | --- | --- | --- | --- | --- |
|  | | | | | | | | | | | | | | | | | | **95% Confidence Interval** | | | |
|  | |  | |  | |  | |  | | **Estimate** | | **Std. Error** | | **z-value** | | **p** | | **Lower** | | **Upper** | |
| age |  | → |  | GM Volume |  | → |  | LTFC |  | 0.324 |  | 0.153 |  | 2.124 |  | 0.034 |  | 0.025 |  | 0.623 |  |
| age |  | → |  | GM Volume |  | → |  | LIFG |  | 0.387 |  | 0.371 |  | 1.042 |  | 0.297 |  | -0.341 |  | 1.115 |  |
| age |  | → |  | GM Volume |  | → |  | LTAFG |  | 0.104 |  | 0.185 |  | 0.562 |  | 0.574 |  | -0.258 |  | 0.465 |  |
| age |  | → |  | GM Volume |  | → |  | SPL |  | 1.051 |  | 0.601 |  | 1.748 |  | 0.080 |  | -0.127 |  | 2.230 |  |
| age |  | → |  | GM Volume |  | → |  | SPL_2 |  | 0.901 |  | 0.534 |  | 1.689 |  | 0.091 |  | -0.145 |  | 1.947 |  |
|  | | | | | | | | | | | | | | | | | | | | | |

GM=grey matter, p=p-value, std=standard.

Supplementary Table 7. Seed-based correlation between parametric effect on age differences of ROIs and mean RT for each competition of context experiments

| Experiment | Brain area | MNI coordinates (mm^3^) | | | Pearson’s r | *P* |
| --- | --- | --- | --- | --- | --- | --- |
|  |  | x | y | z |  |  |
| C1 |  |  |  |  |  |  |
| Context | Left Inferior Frontal Gyrus | -48 | 28 | -20 | -0.183 | 0.271 |
|  | Left Temporal Anterior Fusiform Gyrus | -34 | -2 | -40 | -0.308 | 0.06 |
|  | Left Temporal Fusiform Cortex, Posterior Division | -38 | -28 | -32 | -0.310 | 0.058 |
|  | Superior Parietal Lobe | 8 | -62 | 66 | -0.372 | 0.022* |
|  | Superior Parietal Lobe | 4 | -72 | 52 | -0.393 | 0.015* |
| C2 | | | | | | |
| Context | Left Inferior Frontal Gyrus | -48 | 28 | -20 | -0.450 | 0.005** |
|  | Left Temporal Anterior Fusiform Gyrus | -34 | -2 | -40 | -0.338 | 0.038* |
|  | Left Temporal Fusiform Cortex, Posterior Division | -38 | -28 | -32 | -0.416 | 0.009** |
|  | Superior Parietal Lobe | 8 | -62 | 66 | -0.346 | 0.033* |
|  | Superior Parietal Lobe | 4 | -72 | 52 | -0.525 | 7.163e-4*** |
| C4 | | | | | | |
| Context | Left Inferior Frontal Gyrus | -48 | 28 | -20 | -0.642 | 1.398e-5*** |
|  | Left Temporal Anterior Fusiform Gyrus | -34 | -2 | -40 | -0.340 | 0.037* |
|  | Left Temporal Fusiform Cortex, Posterior Division | -38 | -28 | -32 | -0.566 | 2.132e-4** |
|  | Superior Parietal Lobe | 8 | -62 | 66 | -0.525 | 7.225e-4*** |
|  | Superior Parietal Lobe | 4 | -72 | 52 | -0.613 | 4.211e-5*** |

*C1=context block one, C2=context block 2, C4=context block 4. RT=reaction time. mm3=a cubic millimeter; MNI=Montreal Neurological Institute; TFCE=Threshold-Free Cluster Enhancement.

Supplementary Table 8. Seed-based correlation between parametric effect on age differences of ROIs and mean RT for each competition of dimension experiments

| Experiment | Brain area | MNI coordinates | | | Pearson’s r | *P* |
| --- | --- | --- | --- | --- | --- | --- |
|  |  | x | y | z |  |  |
| D1 |  |  |  |  |  |  |
| Dimension | Right Inferior Parietal Lobule | 44 | -66 | 24 | -0.316 | 0.054 |
|  | Left Middle Temporal Gyrus, Temporooccipital Part | -38 | -60 | 12 | -0.400 | 0.013* |
|  | Left Superior Parietal Lobule | -14 | -56 | 68 | -0.354 | 0.029* |
|  | Left Frontal Orbital Cortex | -42 | 24 | -18 | -0.335 | 0.04* |
| D2 | | | | | | |
| Dimension | Right Inferior Parietal Lobule | 44 | -66 | 24 | -0.408 | 0.011* |
|  | Left Middle Temporal Gyrus, Temporooccipital Part | -38 | -60 | 12 | -0.451 | 0.005* |
|  | Left Superior Parietal Lobule | -14 | -56 | 68 | -0.335 | 0.04* |
|  | Left Frontal Orbital Cortex | -42 | 24 | -18 | -0.396 | 0.014* |
| D4 | | | | | | |
| Dimension | Right Inferior Parietal Lobule | 44 | -66 | 24 | -0.478 | 0.002** |
|  | Left Middle Temporal Gyrus, Temporooccipital Part | -38 | -60 | 12 | -0.420 | 0.009** |
|  | Left Superior Parietal Lobule | -14 | -56 | 68 | -0.429 | 0.007** |
|  | Left Frontal Orbital Cortex | -42 | 24 | -18 | -0.427 | 0.007** |

*D1=dimension block one, D2=dimension block 2, D4=dimension block 4. RT=reaction time; mm3=a cubic millimeter; MNI=Montreal Neurological Institute; TFCE=Threshold-Free Cluster Enhancement.

Supplementary Table 9. Seed-based correlation between parametric effect on age differences of ROIs and mean RT for each competition of feature experiments

| Experiment | Brain area | MNI coordinates | | | Pearson’s r | *P* |
| --- | --- | --- | --- | --- | --- | --- |
|  |  | x | y | z |  |  |
| F1 |  |  |  |  |  |  |
| Feature | Right Precentral Gyrus/Premotor cortex | 30 | -6 | 64 | -0.372 | 0.021* |
|  | Right Precentral Gyrus/Premotor cortex | 32 | -14 | 64 | -0.371 | 0.022* |
|  | Right Precentral Gyrus/Premotor cortex | 32 | -16 | 68 | -0.234 | 0.158 |
|  | Temporal Fusiform Cortex, posterior division | -32 | -40 | -22 | -0.336 | 0.039* |
|  | Right Precentral Gyrus/Premotor cortex | 32 | -20 | 56 | -0.257 | 0.120 |
|  | Juxtapositional Lobule Cortex (formerly known Supplementary Motor Cortex) | 0 | -12 | 54 | -0.223 | 0.178 |
| F2 | | | | | | |
| Feature | Right Precentral Gyrus/Premotor cortex | 30 | -6 | 64 | -0.370 | 0.022* |
|  | Right Precentral Gyrus/Premotor cortex | 32 | -14 | 64 | -0.352 | 0.030* |
|  | Right Precentral Gyrus/Premotor cortex | 32 | -16 | 68 | -0.398 | 0.013* |
|  | Temporal Fusiform Cortex, posterior division | -32 | -40 | -22 | -0.338 | 0.038* |
|  | Right Precentral Gyrus/Premotor cortex | 32 | -20 | 56 | -0.288 | 0.080 |
|  | Juxtapositional Lobule Cortex (formerly known Supplementary Motor Cortex) | 0 | -12 | 54 | -0.290 | 0.077 |
| F4 | | | | | | |
| Feature | Right Precentral Gyrus/Premotor cortex | 30 | -6 | 64 | -0.380 | 0.019* |
|  | Right Precentral Gyrus/Premotor cortex | 32 | -14 | 64 | -0.326 | 0.046* |
|  | Right Precentral Gyrus/Premotor cortex | 32 | -16 | 68 | -0.402 | 0.012* |
|  | Temporal Fusiform Cortex, posterior division | -32 | -40 | -22 | -0.360 | 0.026* |
|  | Right Precentral Gyrus/Premotor cortex | 32 | -20 | 56 | -0.366 | 0.024* |
|  | Juxtapositional Lobule Cortex (formerly known Supplementary Motor Cortex) | 0 | -12 | 54 | -0.292 | 0.075 |

*F1=dimension block one, F2=dimension block 2, F4=dimension block 4. RT=reaction time. mm3=a cubic millimeter; MNI=Montreal Neurological Institute; TFCE=Threshold-Free Cluster Enhancement.

Supplementary Table 10. Correlation coefficients of RT performance of fMRI task and behavioral demographic information

|  | age | edu | BDI-II | MoCA | FAB | WM | C1 | C2 | C4 | D1 | D2 | D4 | F1 | F2 | F4 | R1 | R2 | R4 |
| --- | --- | --- | --- | --- | --- | --- | --- | --- | --- | --- | --- | --- | --- | --- | --- | --- | --- | --- |
| age | — |  |  |  |  |  |  |  |  |  |  |  |  |  |  |  |  |  |
| edu | -0.363* | — |  |  |  |  |  |  |  |  |  |  |  |  |  |  |  |  |
| BDI-II | -0.31 | -0.14 | — |  |  |  |  |  |  |  |  |  |  |  |  |  |  |  |
| MoCA | -0.30 | 0.13 | -0.06 | — |  |  |  |  |  |  |  |  |  |  |  |  |  |  |
| FAB | -0.503** | 0.369* | 0.11 | 0.26 | — |  |  |  |  |  |  |  |  |  |  |  |  |  |
| WM | -0.742** | 0.438** | 0.09 | 0.381* | 0.387* | — |  |  |  |  |  |  |  |  |  |  |  |  |
| C1 | 0.31 | -0.03 | -0.10 | 0.17 | -0.14 | -0.07 | — |  |  |  |  |  |  |  |  |  |  |  |
| C2 | 0.485** | -0.15 | -0.15 | -0.06 | -0.25 | -0.324* | 0.723*** | — |  |  |  |  |  |  |  |  |  |  |
| C4 | 0.10 | 0.06 | -0.02 | 0.22 | -0.10 | 0.02 | 0.82*** | 0.758*** | — |  |  |  |  |  |  |  |  |  |
| D1 | 0.587*** | -0.10 | -0.15 | -0.07 | -0.32 | -0.419** | 0.588*** | 0.794*** | 0.563*** | — |  |  |  |  |  |  |  |  |
| D2 | 0.42** | -0.19 | -0.14 | 0.05 | -0.13 | -0.331* | 0.711*** | 0.829*** | 0.717*** | 0.745*** | — |  |  |  |  |  |  |  |
| D4 | 0.07 | 0.21 | -0.19 | 0.11 | 8.321e -4 | 0.07 | 0.753*** | 0.62*** | 0.755*** | 0.45** | 0.649*** | — |  |  |  |  |  |  |
| F1 | 0.471** | -0.20 | -0.12 | -0.11 | -0.27 | -0.26 | 0.413** | 0.561*** | 0.32 | 0.692*** | 0.401* | 0.30 | — |  |  |  |  |  |
| F2 | 0.471** | -0.17 | -0.24 | -0.12 | -0.31 | -0.325* | 0.546*** | 0.697*** | 0.529*** | 0.617*** | 0.677*** | 0.562*** | 0.475** | — |  |  |  |  |
| F4 | 0.757*** | -0.24 | -0.23 | -0.20 | -0.366* | -0.512** | 0.581*** | 0.721*** | 0.457** | 0.784*** | 0.719*** | 0.407* | 0.543*** | 0.821*** | — |  |  |  |
| R1 | 0.557*** | -0.28 | -0.02 | -0.16 | -0.19 | -0.338* | 0.32 | 0.395*** | 0.15 | 0.576*** | 0.29 | -0.03 | 0.736*** | 0.403* | 0.573*** | — |  |  |
| R2 | 0.519*** | -0.27 | -0.12 | -0.10 | -0.20 | -0.415** | 0.421** | 0.599*** | 0.358* | 0.72*** | 0.62*** | 0.339* | 0.687*** | 0.652*** | 0.647*** | 0.715*** | — |  |
| R4 | 0.675*** | -0.26 | -0.04 | -0.22 | -0.414** | -0.522*** | 0.393* | 0.538*** | 0.28 | 0.68*** | 0.461** | 0.13 | 0.579*** | 0.59*** | 0.715*** | 0.697*** | 0.727*** | — |

* p < .05, ** p < .01, *** p < .001

Supplementary Table 11. Grey matter volume differences between older and young adults

| Cluster Size (mm^3^) |  | MNI Coordinates | | | | | | t-statistic | | P-value TFCE-Corrected | | Brain Region | |  |
| --- | --- | --- | --- | --- | --- | --- | --- | --- | --- | --- | --- | --- | --- | --- |
|  |  | X | | Y | | Z | |  |  |  |  |  |  |  |
| Old > Young | | | | | | | | | | | | | |  |
| 2752 |  | | -34 | | -49 | | -58 | | 2.61 | | 0.013 | | Superior Parietal Lobule | |
| 1186 |  | | -11 | | 33 | | 42 | | 2.34 | | 0.029 | | Superior Frontal Gyrus | |
| 729 |  | | -49 | | -9 | | -22 | | 2.41 | | 0.031 | | Middle Temporal Gyrus | |
| 498 |  | | -59 | | -18 | | 1 | | 2.20 | | 0.035 | | Superior Temporal Gyrus | |
| 135 |  | | 19 | | 53 | | 9 | | 2.37 | | 0.032 | | Frontal pole | |

Local maxima within each significant cluster showing significant (FEW corrected p < 0.05) age differences in grey matter volume. mm3=a cubic millimeter; MNI=Montreal Neurological Institute; TFCE=Threshold-Free Cluster Enhancement.

Supplementary Table 12. Results of group comparison between older and young adults in contrast target trials with null trials during parametric effects of context experiment

| Z-scores | Voxel sizes (mm^3^) | MNI 152 Coordinates (mm^3^) | | | Brain regions (Harvard-Oxford cortical and subcortical structural atlases) |
| --- | --- | --- | --- | --- | --- |
|  |  | x | y | z |  |
| 5.77 | 1343 | -48 | 28 | -20 | Left Inferior Frontal Gyrus |
| 5.42 | 6569 | -34 | -2 | -40 | Left Temporal Anterior Fusiform Gyrus |
| 5.39 | 1527 | -38 | -28 | -32 | Left Temporal Fusiform Cortex, Posterior Division |
| 4.99 | 5718 | 8 | -62 | 66 | Superior Parietal Lobe |
| 4.69 | 5450 | 4 | -72 | 52 | Superior Parietal Lobe |

mm^3^=a cubic millimeter; MNI=Montreal Neurological Institute

Supplementary Table 13. Results of group comparison between older and young adults in contrast target trials with null trials during parametric effects of dimension experiment

| Z-scores | Voxel sizes (mm^3^) | MNI 152 Coordinates (mm^3^) | | | Brain regions (Harvard-Oxford cortical and subcortical structural atlases) |
| --- | --- | --- | --- | --- | --- |
|  |  | x | y | z |  |
| 3.71 | 2062 | 44 | -66 | 24 | Right Inferior Parietal Lobule |
| 3.76 | 3284 | -38 | -60 | 12 | Left Middle Temporal Gyrus, Temporooccipital Part |
| 4.19 | 5693 | -14 | -56 | 68 | Left Superior Parietal Lobule |
| 4.19 | 7320 | -42 | 24 | -18 | Left Frontal Orbital Cortex |

Supplementary Table 14. Results of group comparison between older and young adults in contrast target trials with null trials during parametric effects of feature experiment

| Z-scores | Voxel sizes (mm^3^) | MNI 152 Coordinates (mm^3^) | | | Brain regions (Harvard-Oxford cortical and subcortical structural atlases/Juelich Histological Atlas) |
| --- | --- | --- | --- | --- | --- |
|  |  | x | y | z |  |
| 5.3 | 4512 | 30 | -6 | 64 | Right Precentral Gyrus/Premotor cortex |
| 5.12 | 6896 | 32 | -14 | 64 | Right Precentral Gyrus/Premotor cortex |
| 4.99 | 6973 | 32 | -16 | 68 | Right Precentral Gyrus/Premotor cortex |
| 4.47 | 5590 | -32 | -40 | -22 | Temporal Fusiform Cortex, posterior division |
| 4.45 | 7297 | 32 | -20 | 56 | Right Precentral Gyrus/Premotor cortex |
| 4.45 | 3841 | 0 | -12 | 54 | Juxtapositional Lobule Cortex (formerly known Supplementary Motor Cortex) |

mm3=a cubic millimeter; MNI=Montreal Neurological Institute

Supplementary Table 15. Between-group differences of accuracy in context experiments

| Independent Samples T-Test | | | | | | | | | |
| --- | --- | --- | --- | --- | --- | --- | --- | --- | --- |
|  | | t | | df | | p | | Cohen's d | |
| C1 |  | -3.136 |  | 36 |  | 0.003 |  | -1.018 |  |
| C2 |  | -3.343 |  | 36 |  | 0.002 | ᵃ | -1.085 |  |
| C4 |  | -3.514 |  | 36 |  | 0.001 |  | -1.140 |  |
|  | | | | | | | | | |
| *Note.*  Student's t-test. | | | | | | | | | |

C=context experiment; df=degree of freedom; p=p-value.

Supplementary Table 16. Correlation between working memory capacity and IES score of task performance

| Working memory x IES | | | |
| --- | --- | --- | --- |
|  | Pearson’s r | p | BF_10_ |
| C1 | -0.409 | 0.011* | 4.620 |
| C2 | -0.604 | 5.945e-5*** | 472.292 |
| C4 | -0.163 | 0.328 | 0.321 |
| D1 | -0.460 | 0.004** | 11.891 |
| D2 | -0.502 | 0.001** | 28.815 |
| D4 | 0.104 | 0.534 | 0.243 |
| F1 | -0.269 | 0.103 | 0.728 |
| F2 | -0.307 | 0.047* | 1.100 |
| F4 | -0.611 | 4.611e-5*** | 595.244 |
| R1 | -0.352 | 0.030* | 1.930 |
| R2 | -0.355 | 0.029* | 2.035 |
| R4 | -0.414 | 0.010* | 4.997 |

*p<.05, **p<.01, ***<.001. *IES=inverse efficiency score
